# Polyubiquitylated rice stripe virus NS3 translocates to the nucleus to promote cytosolic virus replication via miRNA-induced fibrillin 2 upregulation

**DOI:** 10.1371/journal.ppat.1012112

**Published:** 2024-03-20

**Authors:** Lu Zhang, Yao Li, Jens H. Kuhn, Kun Zhang, Qisheng Song, Fang Liu

**Affiliations:** 1 College of Plant Protection; Yángzhōu University; Yángzhōu, Jiāngsū Province; China; 2 Integrated Research Facility at Fort Detrick; National Institute of Allergy and Infectious Diseases, National Institutes of Health, Fort Detrick; Frederick, Maryland; United States of America; 3 Division of Plant Science and Technology; College of Agriculture; Food and Natural Resources; University of Missouri; Columbia, Missouri; United States of America; Agriculture and Agri-Food Canada, CANADA

## Abstract

Viruses are encapsidated mobile genetic elements that rely on host cells for replication. Several cytoplasmic RNA viruses synthesize proteins and/or RNAs that translocate to infected cell nuclei. However, the underlying mechanisms and role(s) of cytoplasmic—nuclear trafficking are unclear. We demonstrate that infection of small brown planthoppers with rice stripe virus (RSV), a negarnaviricot RNA virus, results in K63-linked polyubiquitylation of RSV’s nonstructural protein 3 (NS3) at residue K127 by the RING ubiquitin ligase (E3) LsRING. In turn, ubiquitylation leads to NS3 trafficking from the cytoplasm to the nucleus, where NS3 regulates primary miRNA pri-miR-92 processing through manipulation of the microprocessor complex, resulting in accumulation of upregulated miRNA lst-miR-92. We show that lst-miR-92 regulates the expression of fibrillin 2, an extracellular matrix protein, thereby increasing RSV loads. Our results highlight the manipulation of intranuclear, cytoplasmic, and extracellular components by an RNA virus to promote its own replication in an insect vector.

## Author summary

Many DNA and a few RNA viruses use host cell nuclei for viral replication. However, many RNA viruses replicate in the cytoplasm of their host cells but encode proteins that localize to the nuclei or nucleoli. The underlying mechanisms for nuclear localization of viral proteins encoded by cytoplasmic replicating RNA viruses are still largely elusive despite many of these viruses being important plant, animal, and human pathogens. Here, we report that infection of rice stripe virus (RSV), a typical RNA virus transmitted by the small brown planthopper *Laodelphax striatellus*, promotes RSV’s non-structural protein 3 (NS3) nuclear entry via the RING ubiquitin ligase (E3) LsRING catalyzing K63-linked ubiquitination. Once entering the nuclei, NS3 regulates the processing of primary miRNA pri-miR-92 through microprocessor complex to induce accumulation of lst-miR-92. Furthermore, lst-miR-92 positively regulates the expression of fibrillin-2, an extracellular matrix protein, and subsequently increases RSV loads. Our results reveal that virus hijacks ubiquitin-proteasome system in nuclear trafficking of viral nonstructural protein and activates gene transcription through miRNA in insect vector. Our study may provide new ideas for the prevention and/or management of virus disease by regulation of ubiquitin and/or miRNAs.

## Introduction

Viruses are obligate intracellular mobile genetic elements that depend on host cell machinery for replication and genome packaging [1]. Numerous DNA and a few RNA viruses use host cell nuclei for virion formation and/or genome replication [2]. Many RNA viruses—such as paramyxovirids [3,4], coronavirids [5,6], and orthoflaviviruses [7,8] replicate in the cytoplasm of their host cells but encode proteins that localize to the nuclei or nucleoli. Host proteins, such as importin [9] and transportin [10], have been implicated in virion transport across the nuclear envelope. However, the underlying mechanisms for nuclear localization of viral proteins encoded by cytoplasmic replicating RNA viruses are elusive, and consequently their roles in viral lifecycles are unclear. Plant viruses are largely transmitted by hemipteran and thysanopteran insects, i.e., aphids, planthoppers, thrips, and whiteflies [11,12]. Worldwide damage from disease-causing plant viruses has been estimated to be as high as $6 billion annually [13].

Rice stripe virus (RSV) is a global problem for rice agriculture in Asia [14]. RSV is a negarnaviricot (*Bunyavirales*: *Phenuiviridae*: *Tenuivirus oryzaclavatae*) that replicates in and is transmitted by small brown planthoppers (SBPHs; Hemiptera: Delphacidae: *Laodelphax striatellus* (Fallén, 1826)), in a persistent-propagative manner [14]. The RSV genome consists of four linear single-stranded RNA segments. RNA1 (8.9 kb) is negative-sense and encodes an RNA-directed RNA polymerase (RdRp; 337 kDa) [15]. The remaining three segments are ambisense and encode two proteins each: RNA2 (3.5 kb) encodes a nonstructural protein 2 (NS2; 22.8 kDa) and glycoprotein precursor NSvc2 (94.2 kDa); RNA3 (2.4 kb) encodes a nucleocapsid protein (N; 35 kDa) and a gene-silencing suppressor (NS3; 23.9 kDa); and RNA4 (3.5 kb) encodes a disease-specific protein (SP; 21.5 kDa) and a movement protein (NSvc4; 32.5 kDa) [16–18]. RSV replicates in the cytoplasm of infected host cells and is considered a typical representative of cytoplasmic replicating viruses [19–21]. However, N, NS3, and SP localize to host cell nuclei at least in infected benth (*Nicotiana benthamiana* Domin) [22,23]. In SBPHs, N and viral genomic RNAs are transported into host cell nuclei via the importin α nuclear transport system [24].

Our prior research indicated that ubiquitin-proteasome system (UPS) proteins were highly expressed in the salivary glands of RSV-infected SBPHs compared to nonviruliferous controls [25]. Ubiquitin (Ub) is a small, extremely conserved, protein that consists of 76 amino-acid residues and is widely expressed in eukaryotic cells [26]. Ubiquitylation of substrates occurs via a three-step enzymatic cascade that involves a Ub-activating enzyme (E1), a Ub-conjugating enzyme (E2), and a Ub ligase (E3) that catalyze the activation, conjugation, and ligation of one (monoubiquitylation) or several (polyubiquitylation) Ub residues to substrate proteins, respectively [27]. E3s are grouped into three protein families: really interesting new gene (RING), homologous to E6-AP carboxyl terminus (HECT), and RING-between-RING (RBR) [28]. Ubiquitylation may lead to substrate degradation via the UPS, which has important functions in all aspects of cell biology, including autophagy, cell cycle progression, DNA repair, immunity, and protein degradation [27,29]. Following viral infection, the UPS may play multiple roles. For instance, the UPS may degrade ubiquitylated virus proteins, thereby impairing or abolishing virus replication [29]. Conversely, the UPS can be hijacked by a virus to improve its replication [30,31]. The UPS can boost various stages of a virus’s lifecycle through other activities, such as uncoating during virion cell entry [32–35], control of virus protein stability [36], and trafficking and budding of progeny virions [37].

The common mechanism of ubiquitylation involved in virus replication is proteasomal degradation of the substrate by the 26S proteasome in all eukaryotic organisms [26]. Ubiquitylation alone can manipulate virus replication by modulating translocation of viral proteins between host cell nuclei and cytoplasm [3,33] and by stimulating virus polymerase activity [38]. We therefore hypothesized that ubiquitylation and the UPS may be part of the RSV lifecycle. We demonstrate that RSV NS3 enters SBPH nuclei by hijacking the UPS and that polyubiquitylated NS3 subsequently regulates microRNA (miRNA) processing and thereby induces the accumulation of lst-miR-92, which, in turn, upregulates fibrillin 2 and thereby RSV replication.

## Results

### LsRING-mediated ubiquitylation increases RSV loads

Amino-acid sequences of fruit fly (*Drosophila melanogaster* Meigen, 1830) Ub and E3s were used to search the SBPH transcriptome database. Full-length sequences of SBPH Ub-encoding (*LsUb*) and two E3-endoding genes were retrieved. The *LsUb* open reading frame (ORF) is 228 bp long and encodes a predicted 8-kDa protein of 76 amino-acid residues (LsUb, S1A Fig) (GenBank accession no. WDR24501). The LsUb sequence is nearly identical to those of Ubs from a wide variety of animals, ranging from insects to humans (S1A Fig). A total of 114 E3 genes were identified in the SBPH transcriptome database (S1 Table), The RING-type and HECT-type E3 ligases are the classically recognized E3 ubiquitin ligases frequently associated with viral infection [39,40], thus RING type and HECT type were cloned for further study (GenBank accession no. MW751455 and no. MW767162, respectively). The 5,091-bp *LsRING* ORF encodes a protein of 1,697 amino-acid residues containing the RING domain at residues 1,645–1,690 (LsRING; S1B Fig). The 2,739-bp *LsHECT* ORF encodes a protein of 913 amino-acid residues with the HECT domain at residues 582–913 (LsHECT; S1C Fig). LsRING and LsHECT are most similar in amino-acid sequence to NlRING and NlHECT, the RING and HECT E3s of brown planthoppers (*Nilaparvata lugens* (Stål, 1854); GenBank accession no. XP_022198245.1; 86.15% identity [S1B Fig] and GenBank accession no. XP_022187800; 81.24% identity [S1C Fig], respectively).

**Fig 1:**
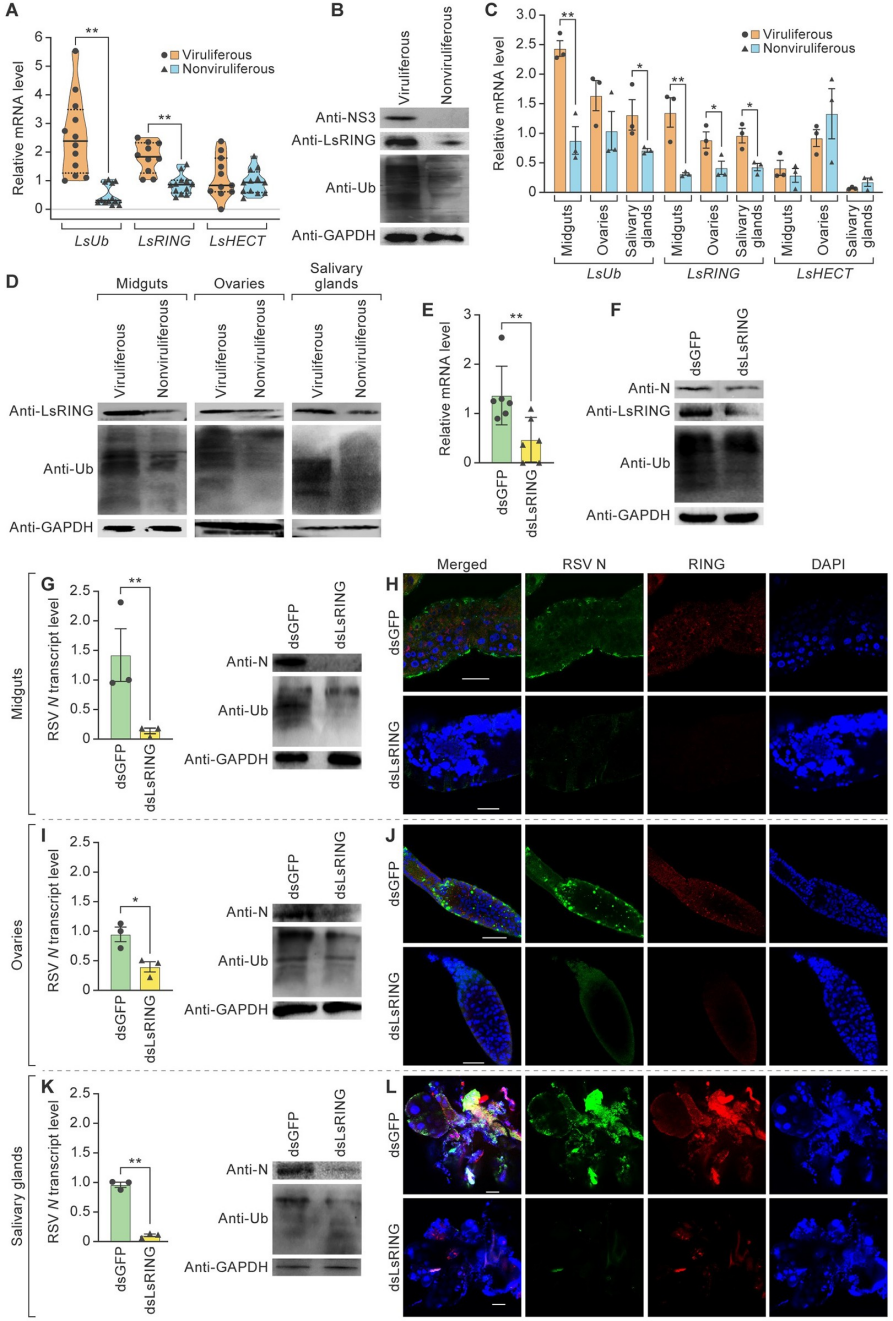
LsRING-mediated ubiquitylation increased RSV loads. (A) Real-time reverse transcription polymerase chain reaction (RT-qPCR) analysis of small brown planthopper (SBPH) ubiquitin (*LsUb*), RING E3 (*LsRING*), and HECT E3 (*LsHECT*) mRNA transcript levels in viruliferous (n = 10) and nonviruliferous (n = 10) SBPHs. (B) Immunoblot analysis of ubiquitylated rice stripe virus (RSV) nonstructural protein 3 (NS3), LsRING, and ubiquitylated proteins in viruliferous (n = 20) and nonviruliferous (n = 20) SBPHs. Glyceraldehyde-3-phosphate dehydrogenase (GAPDH) was used as a loading control. (C) RT-qPCR analysis of LsUb, LsRING, and LsHECT mRNA transcript levels in midguts (n = 300), ovaries (n = 300), and salivary glands (n = 300) of viruliferous and nonviruliferous SBPHs. (D) Immunoblot analysis of GAPDH (loading control), LsRING, and ubiquitylated proteins in midguts (n = 300), ovaries (n = 300), and salivary glands (n = 300) of viruliferous SBPHs and nonviruliferous SBPHs. (E) RT-qPCR analysis of RSV *N* mRNA transcript levels in viruliferous SBPHs treated with dsLsRING (n = 6) or dsGFP (n = 6). (F) Immunoblot analysis of GAPDH (loading control), LsRING, RSV nucleocapsid protein (N), and ubiquitylated proteins in viruliferous SBPHs treated with double-stranded RNA derived from *LsRING* (dsLsRING; n = 20) or *GFP* (dsGFP; n = 20). (G–K) RSV *N* transcript levels in SBPH ovaries (G) (n = 300), midguts (I) (n = 300), and salivary glands (K) (n = 300) determined by RT-qPCR. Levels of ubiquitylated proteins, GAPDH (control), and RSV N were detected by immunoblotting. (H–L) Viruliferous SBPHs were treated with dsLsRING or dsGFP (control) and immunolabeled with anti-RSV N (Alexa Fluor 488; green) or anti-LsRING (Alexa Fluor 555; red) antibodies or stained with 4′,6 diamidino-2-phenylindole (DAPI; blue) and examined by confocal microscopy. Panels: H, midguts (n = 15); J, ovaries (n = 15); and L, salivary glands (n = 15). Scale bar, 50 μm. Abbreviations: mg, midgut; sg, salivary glands; ov, ovary. *p<0.05 and **p<0.01; *t*-test analysis, mean ± standard error of the mean (SEM).

We determined the *LsUb* and *LsE3s* mRNA transcript levels in viruliferous and nonviruliferous SBPHs to investigate their potential roles in RSV infection. Quantitative real-time reverse transcription polymerase chain reaction (RT-qPCR) revealed that *LsUb* was upregulated about 461% in viruliferous SBPHs compared to nonviruliferous SBPHs (Fig 1A). RSV infection also significantly increased *LsRING* but did not affect *LsHECT* (Fig 1A). Furthermore, concentrations of ubiquitylated proteins and LsRING were higher in viruliferous SBPHs relative to nonviruliferous SBPHs (Figs 1B and S2A). Next, we measured the *LsUb*, *LsRING*, and *LsHECT* mRNA transcript levels in midguts, ovaries, and salivary glands by RT-qPCR. The *LsUb* transcript level was significantly higher in the midguts (176%) and salivary glands (84%) of viruliferous vs. nonviruliferous SBPHs, but the difference was not significant in ovaries (Fig 1C). *LsRING* transcription was upregulated in all tissues of viruliferous SBPHs and was highly upregulated in midguts (330%), followed by salivary glands (123%) and ovaries (112%) (Fig 1C). In contrast, RSV infection did not affect transcription of *LsHECT* in different tissues (Fig 1C). Immunoblotting confirmed that LsRING and the ubiquitylated proteins accumulated in higher concentrations in the midguts, ovaries, and salivary glands of viruliferous vs nonviruliferous SBPHs (Figs 1D and S2C). These results indicated that RSV infection may activate the UPS via LsRING.

To further investigate whether LsRING plays a major role in elevated protein ubiquitylation, third instar viruliferous SBPH nymphs were injected with double-stranded RNAs derived from *GFP* (dsGFP) or *LsRING* (dsLsRING) genes. In dsLsRING-treated SBPHs, *LsRING* mRNA transcript levels were reduced by 90% compared to dsGFP-treated controls (S3A Fig). LsRING transcript and protein levels in midguts, ovaries, and salivary glands of dsLsRING-treated SBPHs were consistently lower than those in dsGFP controls (S3B, S3C and S3D Fig), indicating that LsRING knockdown mediated by RNA interference (RNAi) was highly effective. We then investigated whether LsRING knockdown affected protein ubiquitylation. Immunoblotting showed that protein ubiquitylation occurred at lower concentrations in dsLsRING-treated SBPHs than in dsGFP controls (Figs 1F and S2B), indicating that RSV-enhanced LsRING expression is probably involved in protein ubiquitylation.

Next, we compared RSV N expression in SBPHs treated with dsLsRING or dsGFP and found that *N* transcript levels were reduced by 66% in dsLsRING-treated SBPHs compared to dsGFP controls, and the same was true for RSV N expression level (Figs 1E and 1F; S2B). These results indicate that LsRING-mediated ubiquitylation plays a critical role in increasing RSV loads.

The levels and subcellular localization of ubiquitylated proteins, LsRING and RSV loads were then analyzed in midguts, ovaries, and salivary glands of SBPHs treated with dsLsRING or dsGFP (Fig 1G–1L). In midguts, dsLsRING treatment caused a significant reduction in RSV *N* mRNA (decrease of 90%) (Fig 1G, left panel) and reduced the accumulation of RSV ribonucleoprotein complexes (Fig 1H). Protein ubiquitylation levels were much lower in the midguts of dsLsRING-treated SBPHs compared to the dsGFP controls (Figs 1G, right panel; S2D). In ovaries and salivary glands, dsLsRING treatment reduced the accumulation of RSV ribonucleoprotein complexes (Fig 1J and 1L) and N expression (decreases of 58 and 89%, respectively) (Figs 1I and 1K, left panels; S2D). dsLsRING treatment reduced RSV N protein levels in ovaries and salivary glands (Figs 1I and 1K, right panels; S2D), but ubiquitylation levels differed less significantly among dsLsRING treatment and control groups (Figs 1I and 1K, right panels; S2D). These data are consistent with strong upregulation of LsUb and LsRING in the midgut (Fig 1C), where RSV ribonucleoprotein complexes accumulate [41].

### LsRING interacts with RSV NS3

**Fig 2:**
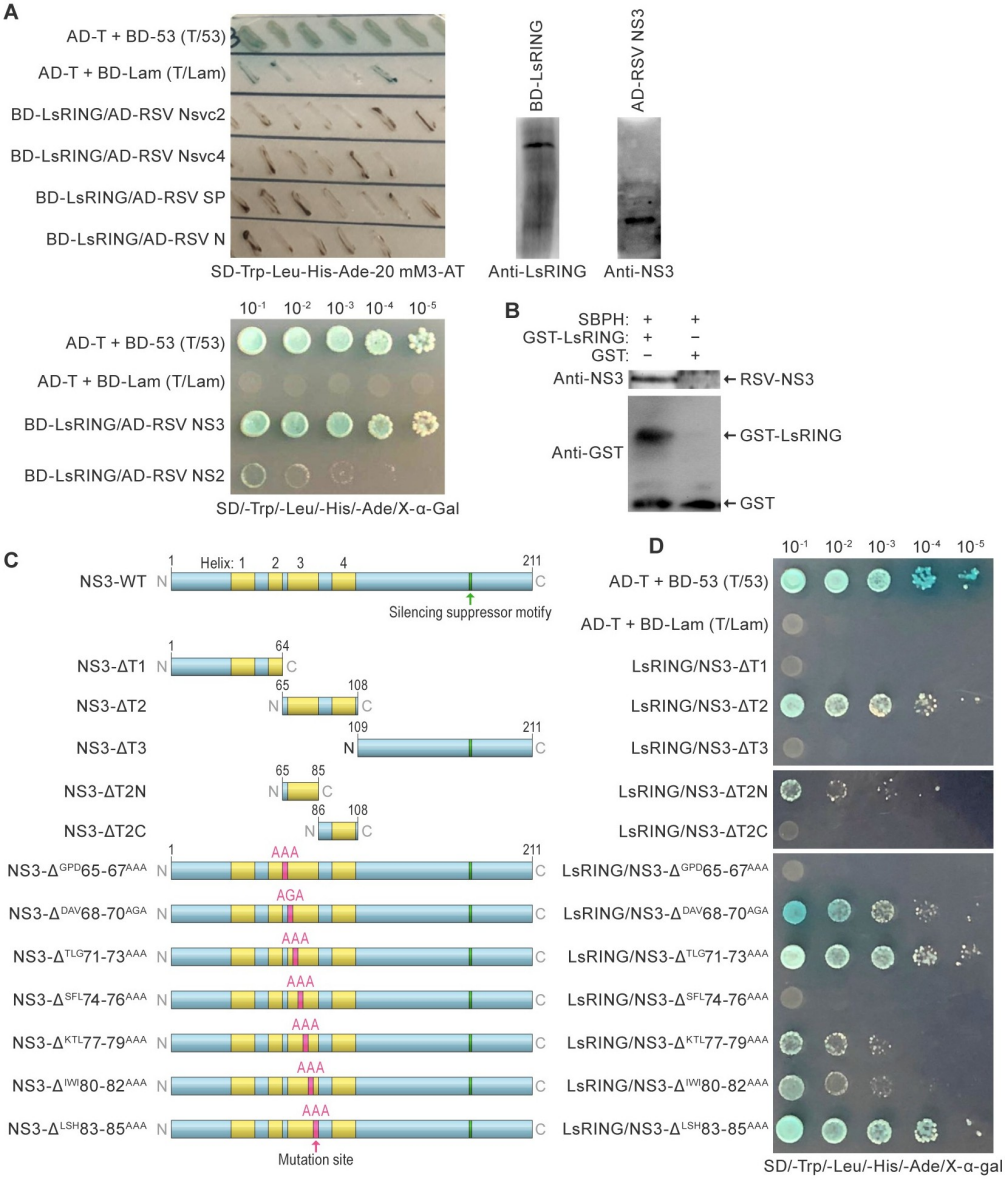
LsRING interacts with RSV NS3. (A) Interaction of small brown planthopper (SBPH) RING E3 (LsRING) and rice stripe virus (RSV) nonstructural protein 3 (NS3) using the yeast two-hybrid assay. Yeast cells were co-transformed with LsRING and RSV NS2, NS3, Nsvc2, Nsvc4, SP, and N. Dilutions of yeast cells were plated onto quadruple-dropout (QDO) SD-Trp-Leu-His-Ade-20 mM3-AT medium, and clones that grew on QDO medium were analyzed for β-galactosidase activity (blue). AD-T + BD-53 (T/53) served as the positive control; AD-T + BD-Lam (T/Lam) served as the negative control. To the right: immunoblot analysis of RSV NS3 and LsRING in yeast. (B) Glutathione *S*-transferase (GST) pull-down assay. GST-LsRING was purified and used as a bait protein; GST was used as a control. Proteins were combined with viruliferous SBPH extracts and incubated with glutathione-*S* beads. Beads were washed to release proteins, which were separated by sodium dodecyl sulfate-polyacrylamide gel electrophoresis (SDS-PAGE). Immunoblots were probed with anti-GST and anti-NS3 antibodies. (C) Schematic diagram of wild-type RSV NS3 and 12 NS3 variants. Five variants were truncated forms of NS3, including residues at positions 1–64 (NS3-ΔT1), 65–108 (NS3-ΔT2), 109–211 (NS3-ΔT3), 65–85 (NS3-ΔT2N), and 86–108 (NS3-ΔT2C). Seven NS3 variants contained alanine substitutions, including substitutions at positions 65–67 (NS3-Δ^GPD^65-67^AAA^), 68–70 (NS3-Δ^DAV^68-70^AGA^), 71–73 (NS3-Δ^TLG^71-73^AAA^), 74–76 (NS3-Δ^SFL^74-76^AAA^), 77–79 (NS3-Δ^KTL^77-79^AAA^), 80–82 (NS3-Δ^IWI^80-82^AAA^), and 83–85 (NS3-Δ^LSH^83-85^AAA^). (D) Interactions of LsRING and NS3 variants using yeast two-hybrid assays. AD-T + BD-53 (T/53) and AD-T + BD-Lam (T/Lam) served as positive and negative controls, respectively.

We hypothesized that LsRING-mediated protein ubiquitylation enhances RSV replication in the SBPH midgut, thereby increasing subsequent RSV loads in different tissues. Therefore, we next investigated whether LsUb and LsRING directly interact with RSV proteins. Yeast two-hybrid (Y2H) assays did not reveal interactions of LsUb and RSV proteins (S4 Fig) but demonstrated that RSV NS3 and NS2 interacted with LsRING (Fig 2A) but not RSV N, nonstructural proteins Nsvc2 and Nsvc4, or disease-specific protein SP (Fig 2A). LsRING bound strongly with NS3 but weakly with NS2 (Fig 2A). We also confirmed the LsRING-NS3 interaction using a glutathione *S*-transferase (GST) pull-down assay (Fig 2B).

RSV NS3 has 211 amino-acid residues. To determine the region of NS3 that interacts with LsRING, we performed Y2H analysis with NS3 deletion variants NS3-ΔT1 (NS3 amino-acid residues 1–64), NS3-ΔT2 (residues 65–108), and NS3-ΔT3 (residues 109–211) (Fig 2C). Growth was only observed in yeast strains harboring NS3-ΔT2 and LsRING (Fig 2D, top panel), indicating that RSV NS3 amino-acid residues 65–108 interact with LsRING. We further analyzed variant NS3-ΔT2 by creating subvariants NS3-ΔT2N (amino-acid residues 65–85) and NS3-ΔT2C (residues 86–108) (Fig 2C); LsRING interacted with RSV NS3-ΔT2N in Y2H assays (Fig 2D, mid panel). We next performed alanine scanning of RSV NS3, introducing variants with LsRING into yeast cells. Amino-acid residue substitutions at positions 65–67 (NS3Δ^GPD^65–67^AAA^) or 74–76 (NS3Δ^SFL^74–76^AAA^) abolished the interaction of RSV NS3 and LsRING (Fig 2D, bottom panel).

NS3 is known to function as an RNA-silencing suppressor [42] and is dependent on amino-acid residues 173–177 [43]. Our results therefore suggest that the LsRING—NS3 interaction has downstream consequences independent of RNA silencing and strongly suggest that RSV NS3 might be a substrate of LsRING for ubiquitylation.

### LsRING-mediated RSV NS3 ubiquitylation increases RSV loads by a mechanism other than 26S proteasome degradation

**Fig 3:**
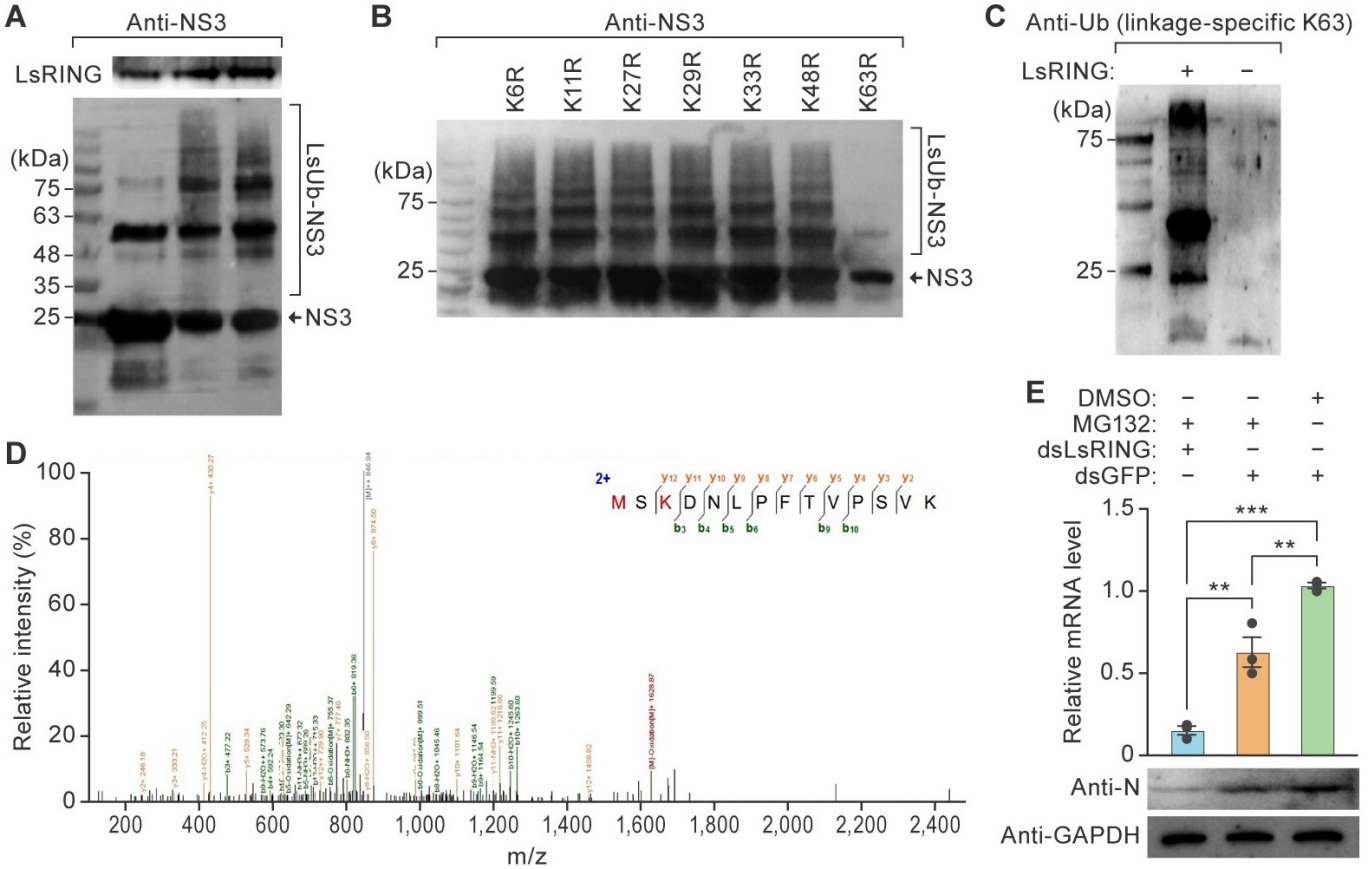
LsRING-mediated RSV NS3 ubiquitylation increases RSV loads by a mechanism other than 26S proteasome degradation. (A) Small brown planthopper (SBPH) RING E3 (LsRING) ubiquitylates rice stripe virus (RSV) nonstructural protein 3 (NS3) *in vitro*. The RSV NS3-His recombinant protein reacted with His-LsRING protein in the presence of E1, E2s, ubiquitin, and adenosine triphosphate (ATP). Immunoblotting analysis was applied to examine the levels of RSV NS3 ubiquitylation by anti-NS3 and the levels of LsRING by anti-LsRING. (B) *In vitro* ubiquitylation of NS3 by LsUb mutants K6 (K6R), K11 (K11R), K27 (K27R), K29 (K29R), K33 (K33R), K48 (K48R), and K63 (K63R). (C) Analysis of the NS3 ubiquitylation site by immunoblotting with a K63 linkage-specific polyubiquitin antibody. (D) Liquid chromatography—mass spectrometry (LC–MS)/MS analysis of the NS3 ubiquitylation site. Following the *in vitro* ubiquitylation assay, NS3 was resolved by sodium dodecyl sulfate-polyacrylamide gel electrophoresis (SDS-PAGE), and the uppermost ubiquitylated protein band was excised for LC–MS/MS. K127 is the ubiquitylation site. Peptide sequence and predicted b and y ions are shown. (E) Real-time reverse transcription polymerase chain reaction (RT-qPCR) analysis of the RSV N transcript abundancy in SBPHs treated with MG132 + double-stranded RNA derived from LsRING (dsLsRING; n = 10), MG132 + double-stranded DNA derived from *GFP* (dsGFP; n = 10), and dimethyl sulfoxide (DMSO) + dsGFP (n = 10). *p<0.05 and **p<0.01, *t*-test analysis; mean ± standard error of the mean (SEM). Immunoblot analysis of RSV N and glyceraldehyde-3-phosphate dehydrogenase (GAPDH) protein concentrations in SBPHs treated with MG132 + dsLsRING (n = 10), DMSO + dsLsRING (n = 10), and MG132 + dsGFP (n = 10).

An *in vitro* ubiquitylation assay was conducted to confirm the substrate of LsRING for ubiquitylation. Ub possesses seven lysine residues (K6, K11, K27, K29, K33, K48, and K63) that are known targets for the formation of ubiquityl chains [27]. To identify the lysyl targets of LsRING for RSV NS3 polyubiquitylation, seven LsUb variants were generated: K6 (K6R), K11 (K11R), K27 (K27R), K29 (K29R), K33 (K33R), K48 (K48R), and K63 (K63R). To test if the recombinant LsRING was active, an auto-ubiquitylination assay was performed and the immunoblots against ubiquitin were analyzed. The ubiquitin laddering pattern was seen above the size of ubiquitin (S5 Fig), thus confirming the ubiquitin ligase activity of the recombinant LsRING. LsRING promoted ubiquitylation of RSV NS3 in a concentration-dependent manner (Fig 3A). *In vitro* ubiquitylation assays showed that polyubiquitylation occurred on K6, K11, K27, K29, K33, and K48 variants but not the K63 variant (Fig 3B), revealing that LsRING conjugates K63-linked ubiquityl chains to NS3. The specificity of the K63-specific polyubiquitylation site was confirmed in immunoblots with K63 linkage-specific polyubiquityl antisera (Fig 3C). Furthermore, liquid chromatography—mass spectrometry (LC–MS)/MS analysis identified K127 in RSV NS3 as the residue involved in LsUb K63-linked ubiquitylation (Fig 3D).

The ubiquityl-conjugating system tags ubiquitylated proteins for degradation by the 26S proteasome, and Ub-K48 linkages are well-known proteasome-targeting signals [44]. However, LsRING-mediated RSV NS3 ubiquitylation mainly at K63, implying that LsRING-mediated NS3 ubiquitylation might increase RSV loads by a mechanism other than degradation by the 26S proteasome. To test this hypothesis, MG132, a specific inhibitor of the 26S proteasome, was evaluated for its effect on LsRING-mediated RSV loads. Combined treatment with MG132 and dsGFP reduced RSV *N* transcript levels in viruliferous SBPHs by 39% compared to treatment with dimethyl sulfoxide (DMSO) and dsGFP (Fig 3E). However, combined treatment of MG132 and dsLsRING reduced RSV *N* transcript abundancy in viruliferous SBPHs by 85% compared to the control group (Fig 3E), indicating that the silence of LsRING decreases RSV loads. Immunoblots confirmed that RSV *N* accumulated at lower levels when SBPHs were treated with both MG132 and dsLsRING compared to MG132 and dsGFP dual treatment (Figs 3E and S2E). Collectively, these results imply that LsRING-mediated RSV NS3 ubiquitylation increases RSV loads through a mechanism other than degradation by the 26S proteasome.

### LsRING-mediated RSV NS3 ubiquitylation mediates NS3 translocation into the nucleus

**Fig 4:**
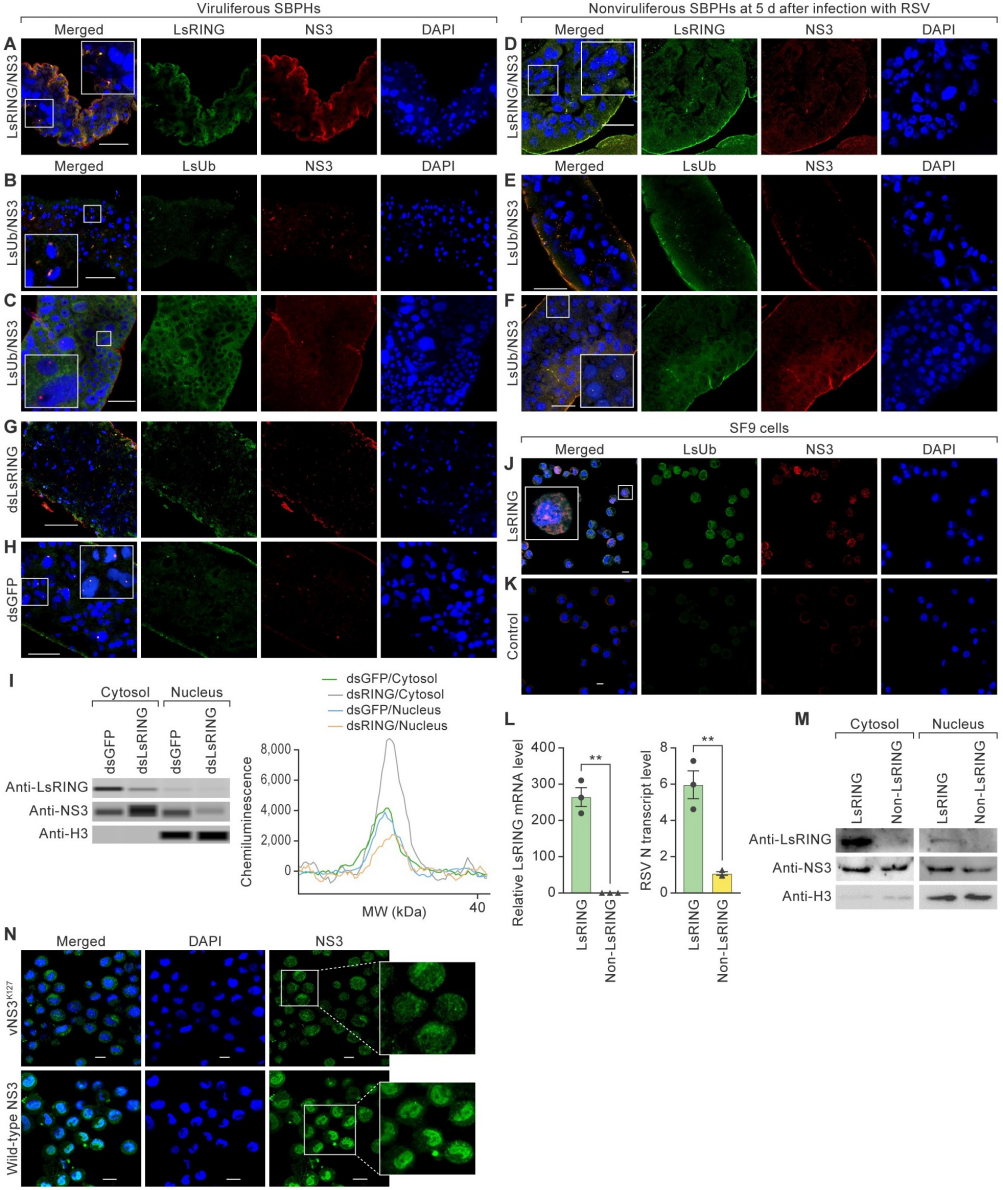
LsRING-mediated RSV NS3 ubiquitylation mediates NS3 translocation into the nucleus. Small brown planthopper (SBPH) RING E3 (LsRING) and ubiquitin (LsUb) were labeled with antibodies conjugated with Alexa Fluor 488 (green), and rice stripe virus (RSV) nonstructural protein 3 (NS3) was labeled with antisera conjugated with Alexa Fluor 555 (red) [11]. Nuclei were stained with 4′,6 diamidino-2-phenylindole (DAPI; blue). (A) LsRING and NS3 co-localize near the nuclei of midgut cells (n = 20) of viruliferous SBPHs. Scale bar, 50 μm. (B) Representative images of LsUb and NS3 near the nuclei of midgut cells (n = 20) of viruliferous SBPHs. Scale bar, 50 μm. (C) Representative images of LsUb and NS3 in the nuclei of midgut cells (n = 20) of viruliferous SBPHs. Scale bar, 50 μm. (D) LsRING and NS3 co-localize near the nuclei of midgut cells (n = 20) in nonviruliferous SBPHs 5 d after access to diseased plants. Scale bar, 50 μm. (E and F). LsUb and NS3 co-localize around (E) or in (F) the nuclei of midgut cells (n = 20) of nonviruliferous SBPHs at 5 d. Scale bar, 50 μm. (G and H) Representative images of ubiquitylated NS3 entry into the nucleus of midguts from viruliferous SBPHs treated with double-stranded RNAs derived from *LsRING* (dsLsRING; n = 15) or double-stranded *GFP* (dsGFP; n = 15). Scale bar, 50 μm. (I) Analysis of LsRING, NS3, and histone H3 proteins in nuclei and cytoplasm of midgut cells. Proteins were detected by Wes in viruliferous SBPHs treated with dsLsRING or dsGFP. (J–K) NS3 localization in fall armyworm Sf9 cells stably expressing LsRING and eGFP at 24 h after RSV infection. Scale bar, 10 μm. (L) Real-time reverse transcription polymerase chain reaction (RT-qPCR) analysis of *LsRING* and RSV *N* transcript levels in Sf9 cells transfected with LsRING or eGFP expression plasmids. *p<0.05 and **p<0.01, *t*-test analysis; mean ± standard error of the mean (SEM). (M) Immunoblot analysis of LsRING, NS3, and histone H3 proteins in nuclei and cytoplasm of Sf9 cells transfected with LsRING or eGFP expression plasmids 24 h after RSV infection. (N) Detection of NS3 and vNS3^K127^ nuclear entry in Sf9 cells. Scale bar, 10 μm. Note: White-bordered rectangles indicate enlargements of inset panels.

We used immunofluorescence microscopy to determine the subcellular localization of RSV NS3 and SBPH LsUb and LsRING in the midguts of viruliferous SBPHs. NS3 and LsRING (Fig 4A), as well as NS3 and LsUb (Fig 4B), were frequently observed near the nuclei of midgut cells in viruliferous SBPHs. In addition, the co-localizations of NS3 and LsUb were frequently observed in the nuclei of midgut cells in viruliferous SBPHs (Fig 4C). Similar results were observed in midgut cells of nonviruliferous SBPHs 5 d after access to diseased plants (Fig 4D–4F). Since K63-linked ubiquitylation has been implicated in intracellular trafficking [45–47], we hypothesized that LsRING-mediated ubiquitylation of NS3 might facilitate NS3 cytoplasm-nuclear trafficking. This hypothesis was tested by evaluating the localization of LsUb and NS3 in the midguts of viruliferous SBPHs treated with dsGFP or dsLsRING. In dsGFP-treated controls, NS3 co-localized with LsUb around or in cell nuclei; however, in the dsLsRING-treated group, NS3 was primarily present in the cytoplasm (Fig 4G and 4H; S2 Table). These results were confirmed by Wes (Fig 4I), indicating that LsRING-mediated NS3 ubiquitylation promotes RSV NS3 nuclear entry. In fall armyworm (*Spodoptera frugiperda* (J. E. Smith, 1797)) Sf9 cells stably expressing LsRING (produced from a baculovirus expression system), a strong NS3 fluorescent signal was present in nuclei 24 h after RSV exposure (Fig 4J); such a signal was not observed in Sf9 cells stably expressing enhanced GFP (eGFP) (Fig 4K). RT-qPCR showed that the *LsRING* transcript level increased by 3153% and the RSV *N* transcript level increased by 451% (Fig 4L). In nuclei, immunoblot analysis revealed that NS3 level in Sf9 cells overexpressing LsRING was higher than in controls (Figs 4M and S2F). Since LsRING mediates RSV NS3 K127 polyubiquitylation, we generated variant NS3^K127^ (vNS3^K127^) and compared the nuclear-location abilities of vNS3^K127^ and wild-type NS3 using an immunofluorescence. As shown in Figs 4N and S6, a few NS3 mutants that cannot be ubiquitylated can enter the nucleus, and wild-type NS3 nuclear localization was more pronounced.

Taken together, these results demonstrate that LsRING-mediated RSV NS3 ubiquitylation facilitates NS3 nuclear translocation and thereby RSV replication.

### RSV NS3 enters Sf9 cell nuclei 12.5 h after virus exposure

**Fig 5:**
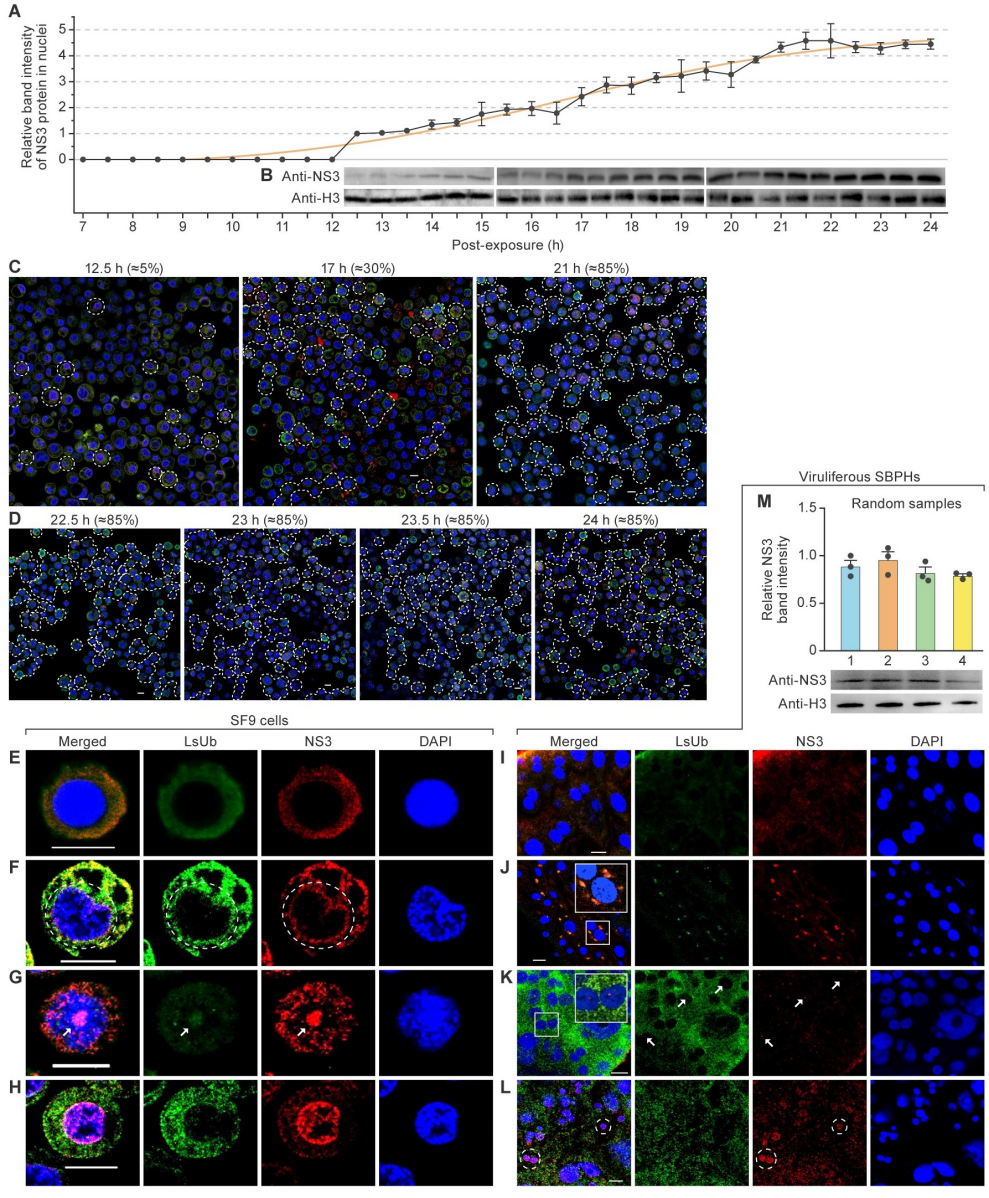
RSV NS3 enters Sf9 cell nuclei 12.5 h after virus exposure. (A) Quantitative analysis of rice stripe virus (RSV) nonstructural protein 3 (NS3) and histone H3 band intensity by ImageJ. (B) Immunoblot analysis of rice stripe virus (RSV) NS3 and histone H3 in fall armyworm Sf9 nuclei at 30-min intervals. Proteins were monitored 7–24 h after RSV infection. Panels C and D show results after small brown planthopper (SBPH) NS3 and ubiquitin (LsUb) were labeled with antibodies conjugated to Alexa Fluor 488 (green) and Alexa Fluor 555 (red) [11], respectively, and nuclei were stained with 4′,6 diamidino-2-phenylindole (DAPI; blue). Scale bar, 10 μm. White dotted lines encircle Sf9 cells containing NS3 in the nuclei. (C) Abundance of NS3 in the nuclei of Sf9 cells at 12.5, 17, and 21 h after RSV infection. (D) Abundance of NS3 in Sf9 nuclei at 22.5, 23, 23.5, and 24 h after RSV infection of Sf9 cells. Panels E–H show representative images demonstrating NS3 and LsUb spatial patterns in Sf9 cells labeled for NS3 (red) [11] and LsUb (green). Scale bar, 10 μm. (E) Co-localization of NS3 and LsUb in the cytoplasm of Sf9 cells. (F) Co-localization of NS3 and LsUb near the perinuclear regions of Sf9 cells. (G) Images of NS3 and Ub in Sf9 nuclei in a dot-like matrix. (H) Images of Sf9 nuclei containing large amounts of NS3. Panels I–L show NS3 and LsUb spatial patterns in SBPH midgut cells labeled for NS3 [11] and LsUb (green). Scale bar, 10 μm. (I) NS3 and LsUb co-localize in the cytoplasm of midgut cells of the viruliferous SBPHs. (J) NS3 and LsUb co-localize near perinuclear regions of midgut cells of viruliferous SBPHs. (K) NS3 and LsUb accumulate in midgut nuclei of viruliferous SBPHs as a circular matrix. (L) Midgut nuclei of viruliferous SBPHs contain large amounts of NS3. (M) NS3 accumulation in midgut nuclei from four randomly selected viruliferous SBPHs; band intensities were determined by ImageJ. Immunoblot analysis of RSV NS3 and histone H3 in the midgut nuclei of four randomly-selected the viruliferous SBPHs. *p<0.05 and **p<0.01; *t*-test analysis; mean ± standard error of the mean (SEM). Note: White-bordered rectangles indicate enlargements of inset panels. White arrowheads indicate distinct NS3 puncta near SBPH nuclei. White dotted lines encircle nuclei.

To determine the temporal pattern of RSV NS3 nuclear entry, Sf9 cells were collected 7 h after RSV infection at 30-min intervals followed by immunoblot using an anti-NS3 antibody. RSV NS3 was undetectable in the nuclei of Sf9 cells 7–11.5 h after RSV exposure. Beginning at 12.5 h, NS3 was increasingly detected in the nuclei (Fig 5A–5C). At 17 and 21 h after RSV exposure, NS3 fluorescent signals were detected in 30% and 85% of nuclei, respectively (Fig 5C). This signal remained relatively stable until 22.5–24 h after RSV exposure (Fig 5D). Moreover, four different spatial patterns were observed in Sf9 cells: (1) LsUb and NS3 dispersed in the cytosol (Fig 5E); (2) LsUb and NS3 co-localized in the perinuclear regions (Fig 5F); (3) nuclear co-localization of LsUb and NS3 resulted in a dot-like matrix (Fig 5G); and (4) high levels of NS3 accumulation occurred in the nuclei (Fig 5H).

The subcellular localization of RSV NS3 was further investigated *in vivo* using randomly collected adult SBPHs that were produced by viruliferous SBPH nymphs. Similar to Sf9 cells, four spatial distribution patterns were observed in the midguts of viruliferous SBPHs: (1) LsUb and NS3 dispersed in the cytosol of SBPH midgut cells (Fig 5I); (2) LsUb and NS3 co-localized near the perinuclear regions (Fig 5J); (3) co-localization of NS3 and LsUb fluorescent signals in the nuclei of midgut cells resulted in a dot-like matrix (Fig 5K); and (4) accumulation of NS3 occurred in the nuclei of midgut cells (Fig 5L). NS3 concentrations were relatively consistent in the nuclei of midgut cells from viruliferous SBPHs (Fig 5M). In conclusion, NS3 localization was similar in Sf9 cells and SBPH midgut tissues.

### RSV NS3 facilitates nuclear processing of primary miRNA (pri-miRNA) and induces accumulation of miRNAs in the cytoplasm

**Fig 6:**
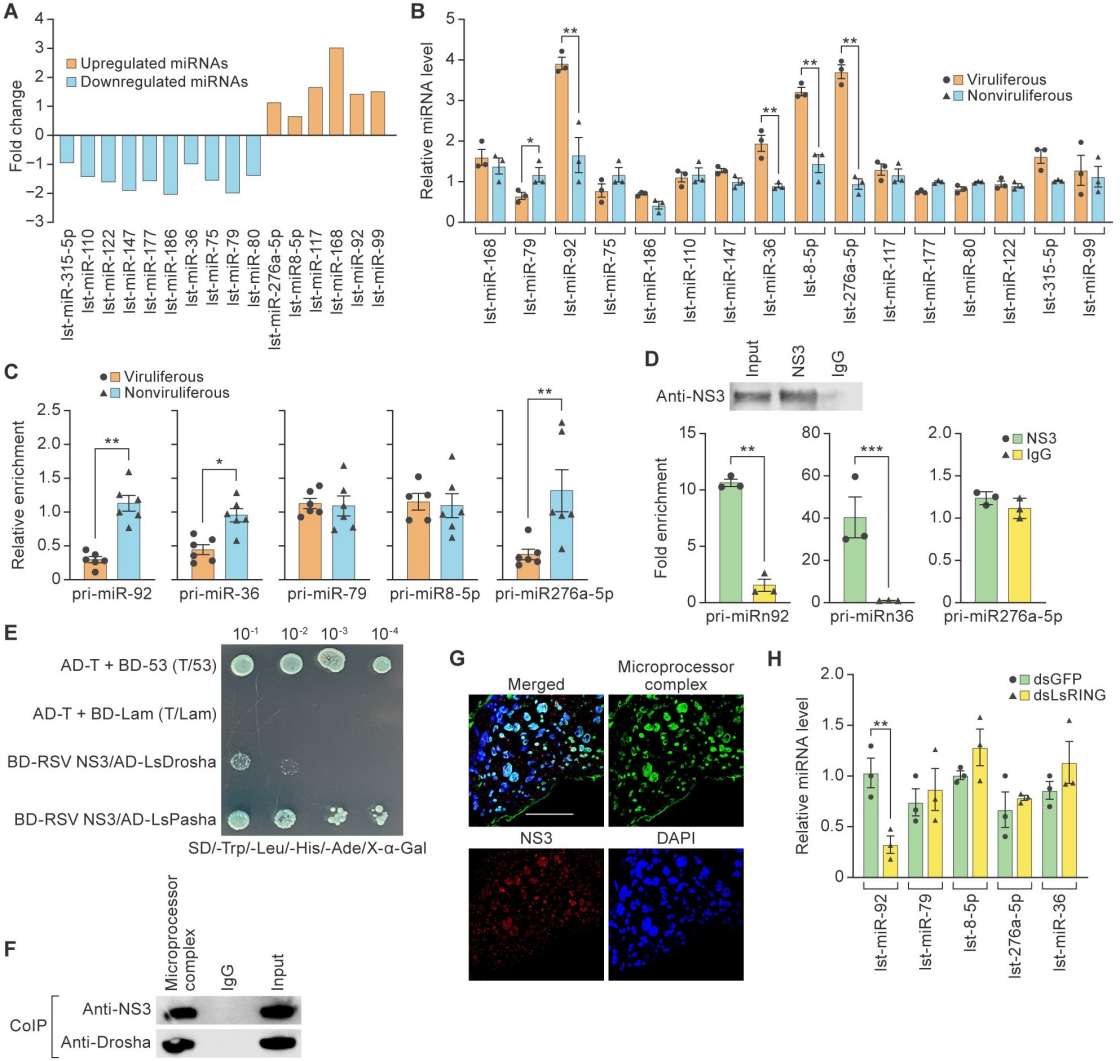
RSV NS3 facilitates nuclear processing of primary miRNA (pri-miRNA) and induces accumulation of miRNAs in the cytoplasm. (A) Differentially expressed (*p*-value < 0.005) microRNAs (miRNAs) of small brown planthoppers (SBPHs) in response to rice stripe virus (RSV) infection. Columns below and above the x axis represent downregulated and upregulated miRNAs, respectively. (B) Real-time reverse transcription polymerase chain reaction (RT-qPCR) analysis of differentially regulated miRNAs in response to RSV infection. (C) RT-qPCR analysis of primary miRNA (pri-miRNA) precursor numbers 92, 36, 79, 8-5p, and 276a-5p in viruliferous (n = 15) and nonviruliferous (n = 15) SBPHs. (D) Immunoprecipitation of RSV nonstructural protein 3 (NS3) targeted and non-target-associated pri-miRNAs. RNA immunoprecipitation (RIP) lysates were prepared from SBPHs and immunoprecipitated using an anti-NS3 antibody or immunoglobulin G (IgG; control) and the RIP kit. (E) Yeast two-hybrid results showing the RSV NS3 interaction with SBPH Drosha (LsDrosha) or Pasha (LsPasha). Yeast cells were co-transformed with plasmids expressing RSV NS3 and LsDrosha and LsPasha. Dilutions of yeast cells were plated onto QDO SD-Trp-Leu-His-Ade-20 mM3-AT medium, and clones that grew on QDO medium were analyzed for β-galactosidase activity (blue). AD-T + BD-53 (T/53) served as the positive control; AD-T + BD-Lam (T/Lam) served as the negative control. (F) Co-immunoprecipitation (CoIP) analysis shows the interaction between NS3 and the SBPH microprocessor complex. Commercial anti-Drosha antibodies were used for the assay because of the overall conserved Drosha sequence (S7 Fig), and IgG was used as the control. (G) Co-localization of NS3 (red) [11] and the microprocessor complex (green) in the nuclei of viruliferous SBPHs. Scale bar, 50 μm. (H) RT-qPCR analysis of lst-miR-92, lst-miR-79, lst-miR-8-5p, lst-miR-276a-5p, and lst-miR-36 in viruliferous SBPHs treated with double-stranded RNA derived from *LsRING* (dsLsRING) or *GFP* (dsGFP; n = 20). *p<0.05 and **p<0.01, *t*-test analysis; mean ± standard error of the mean (SEM).

RSV NS3 overexpression increases the accumulation of miRNAs by promoting pri-miRNA processing in Asian rice (*Oryza sativa* L.) nuclei [48]. Given our observation that NS3 entered the nuclei of SBPH cells (Fig 5), we hypothesized that NS3 may facilitate pri-miRNA processing and affect miRNA accumulation. To test this hypothesis, we sequenced small RNAs in viruliferous and nonviruliferous SBPHs to identify differentially transcribed miRNAs that may be associated with RSV infection. Six differentially upregulated miRNAs (lst-miR-168, lst-miR-92, lst-miR-99, lst-miR-276a-5p, lst-miR-8-5p, and lst-miR-117) and 10 downregulated miRNAs (lst-miR-79, lst-miR-75, lst-miR-186, lst-miR-110, lst-miR-177, lst-miR-80, lst-miR-36, lst-miR-315-5p, lst-miR-122, and lst-miR-147) were identified in viruliferous versus nonviruliferous SBPHs (*p*<0.005) (Fig 6A). Synthesis of these 16 miRNAs was examined in viruliferous and nonviruliferous SBPHs. Four miRNAs (lst-miR-92, lst-miR-36, lst-miR-8-5p, and lst-miR-276a-5p) were upregulated and one miRNA (lst-miR-79) was downregulated in response to RSV infection (Fig 6B). Mature miRNAs are generated in a two-step process: nuclear processing of pri-miRNA by Drosha to produce pre-miRNAs, and cytoplasmic processing of pre-miRNA by Dicer to generate the mature miRNA [49]. To determine whether RSV infection increases accumulation of these miRNAs through promoting pri-miRNA processing, we measured the primary transcript levels of lst-miR-92, lst-miR-36, lst-miR-8-5p, lst-miR-276a-5p, and lst-miR-79 in viruliferous and nonviruliferous SBPHs by RT-qPCR. RSV infection downregulated three pri-miRNAs (lst-miR-92, lst-miR-36, and lst-miR-276a-5p) (Fig 6C), suggesting that RSV infection induces pri-miRNA processing. To further investigate whether RSV infection reduces pri-miRNA accumulation through NS3-mediated pri-miRNA processing, RNA immunoprecipitation (RIP) PCR assays were conducted with anti-NS3 and negative control immunoglobulin G (IgG). RIP-PCR showed that pri-miR-92 and pri-miR-36 were recognized by NS3 (Fig 6D), indicating that NS3 increases the accumulation of lst-miR-92 and lst-miR-36 through pri-miRNA processing and the accumulation of lst-miR-276a-5p through a pri-miRNA-independent mechanism.

RSV NS3 does not contain an RNase III domain and therefore cannot process pri-miRNAs [50]; thus, we hypothesized that NS3 regulates miRNA processing through the microprocessor complex[50]. Indeed, Y2H assays demonstrated that RSV NS3 interacted with components of the microprocessor complex, LsDrosha and LsPasha (Fig 6E). RSV NS3 bound strongly to LsPasha but weakly to LsDrosha (Fig 6E). We further examined the interaction between RSV NS3 and the microprocessor complex. Co-immunoprecipitation (CoIP) assays confirmed that NS3 interacts with the microprocessor complex in viruliferous SBPHs (Fig 6F). Immunofluorescence microscopy results showed that the microprocessor complex and NS3 co-localize in nuclei of midgut cells of viruliferous SBPHs (Fig 6G).

Since LsRING-mediated NS3 ubiquitylation promotes NS3 nuclear translocation and NS3 mediates processing of pri-miRNA in the nucleus, we investigated whether LsRING influences the expression of lst-miR-92, lst-miR-36, lst-miR-8-5p, lst-miR-275a-5p, or lst-miR-79. Interestingly, lst-miR-92 was significantly downregulated by dsLsRING treatment compared to the dsGFP-treated control group (Fig 6H); therefore, we focused on lst-miR-92 in subsequent experiments. Collectively, these results indicate that RSV NS3 facilitates pri-miRNA processing in nuclei, where it uses the microprocessor complex to induce the accumulation of corresponding mature miRNAs.

### RSV NS3 induces fibrillin 2 accumulation by upregulating lst-miR-92 in the cytoplasm to enhance RSV replication

**Fig 7:**
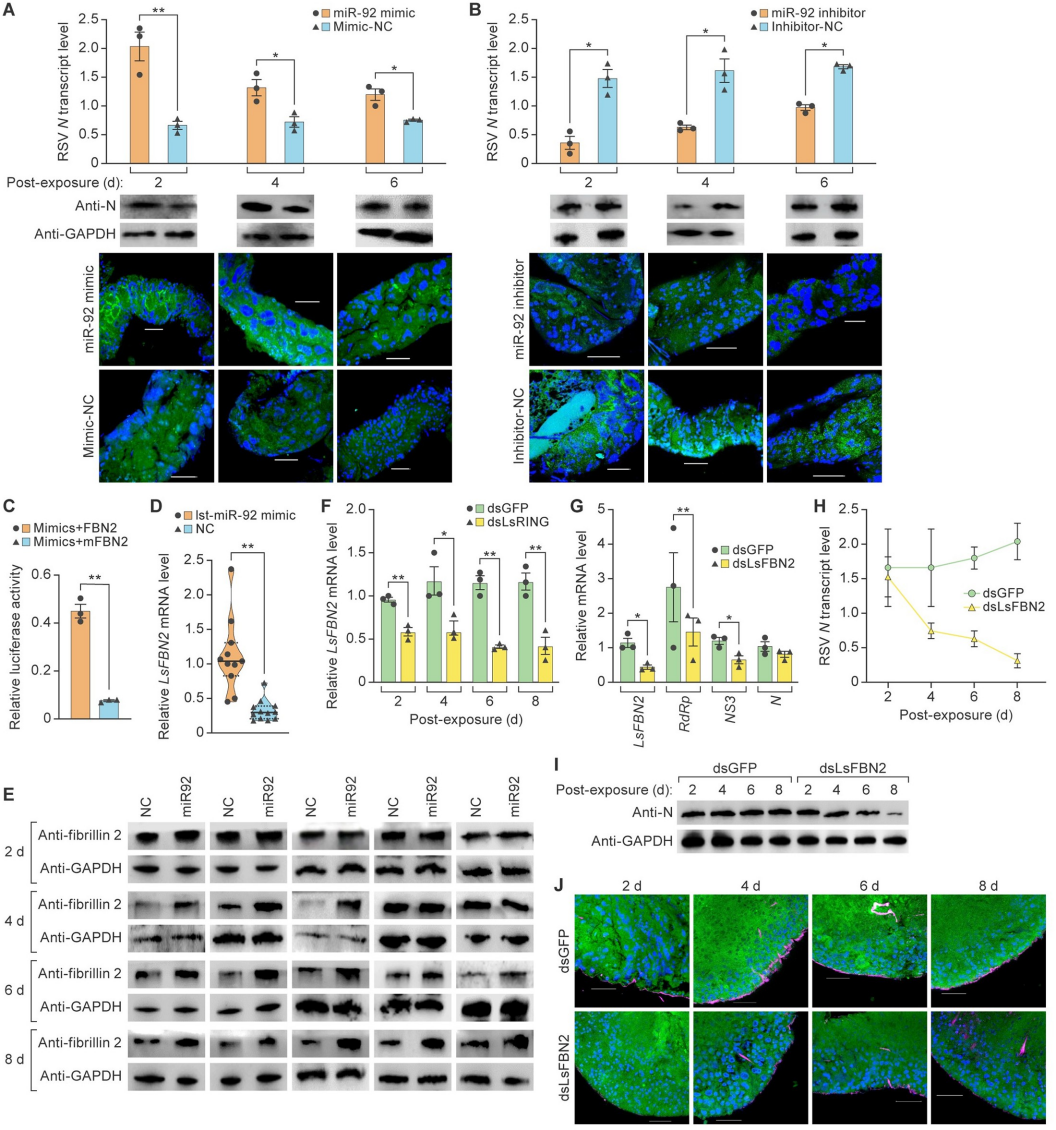
RSV NS3 induces fibrillin 2 accumulation by upregulating lst-miR-92 in the cytoplasm to enhance RSV replication. (A) Real-time reverse transcription polymerase chain reaction (RT-qPCR) analysis of rice stripe virus (RSV) *N* transcript levels in viruliferous SBPHs at 2 (n = 15), 4 (n = 15), and 6 (n = 15) d after miR-92 mimic or mimic negative control (mimic-NC) treatment. Immunoblot analysis of RSV nucleocapsid protein (N) and glyceraldehyde-3-phosphate dehydrogenase (GAPDH) in viruliferous SBPHs at 2 (n = 15), 4 (n = 15), and 6 (n = 15) d after miR-92 mimic or mimic-NC treatment. Viruliferous SBPHs were treated with miR-92 mimic (mimic-NC as a control) and immunolabeled with anti-RSV N antibody (Alexa Fluor 488; green) or stained with 4′,6 diamidino-2-phenylindole (DAPI; blue) before being examined by confocal microscopy. Scale bar, 50 μm. (B) RT-qPCR analysis of RSV *N* transcript levels in viruliferous SBPHs at 2 (n = 15), 4 (n = 15), and 6 (n = 15) d after miR-92 inhibitor or inhibitor-NC treatment. Immunoblot analysis of RSV N and GAPDH in viruliferous SBPHs at 2 (n = 15), 4 (n = 15), and 6 (n = 15) d after miR-92 inhibitor or inhibitor-NC treatment. Viruliferous SBPHs were treated with miR-92 inhibitor (inhibitor-NC as a control) and immunolabeled with anti-RSV N antibody (Alexa Fluor 488; green) or stained with DAPI (blue) before being examined by confocal microscopy. Scale bar, 50 μm. (C) Dual-luciferase assays in Sf9 cells transfected with cloned FBN2 vector or mutant FBN2 (mFBN2) vector and lst-miR-92 mimic. (D) RT-qPCR analysis of *LsFBN2* transcript levels in viruliferous SBPHs treated with lst-miR-92 mimic (n = 12) or negative control (NC; n = 12). (E) Immunoblot analysis of fibrillin 2 and GAPDH in nonviruliferous SBPHs at 2 (n = 50), 4 (n = 50), 6 (n = 50), and 8 (n = 50) d after miR-92 mimic or mimic-NC treatment. (F) RT-qPCR analysis of *LsFBN2 t*ranscript levels in viruliferous SBPHs at 2 (n = 12), 4 (n = 12), 6 (n = 12), and 8 (n = 12) d after dsGFP or dsLsRING treatment. (G) RT-qPCR analysis of *LsFBN2* and RSV RNA-directed RNA polymerase (*RdRp*), *NS3*, and *N* mRNA levels in viruliferous SBPH injected with double-stranded RNA derived from double-stranded *GFP* (dsGFP; n = 12) or *LsFBN2* (dsLsFBN2; n = 12). (H) RT-qPCR analysis of RSV *N* transcript abundancy in viruliferous SBPHs at 2 (n = 15), 4 (n = 15), 6 (n = 15), and 8 (n = 15) d after dsGFP or dsLsFBN2 treatment. (I) Immunoblot analysis of RSV N and GAPDH in viruliferous SBPHs at 2 (n = 15), 4 (n = 15), 6 (n = 15), and 8 (n = 15) d after dsGFP or dsLsFBN2 treatment. (J) Viruliferous SBPHs were treated with dsGFP (control) or dsLsFBN2 and immunolabeled with anti-RSV N antibody (Alexa Fluor 488; green) and anti-LsFBN2 antibody (Alexa Fluor 555; red) or stained with DAPI (blue) before being examined by confocal microscopy. *p<0.05 and **p<0.01, *t*-test analysis; mean ± standard error of the mean (SEM).

Since host miRNAs may affect virus replication [51], we injected third instar viruliferous SBPH nymphs with lst-miR-92 mimic (artificially synthesized lst-miR-92 sequence) and mimic-NC (artificially synthesized non-functional sequence, the negative control) to investigate the function of lst-miR-92 on RSV replication. lst-miR-92 inhibitor (artificially synthesized lst-miR-92 inhibitor sequence) and inhibitor-NC artificially synthesized non-functional sequence, the negative control) treatments were used for further investigation. RT-qPCR analysis showed that lst-miR-92 mimic upregulated the transcript level of RSV RdRp in SBPHs at 2 d and 4 d after lst-miR-92 mimic treatment by 206% and 195%, respectively (S8A Fig). lst-miR-92 inhibitor reduced the transcript level of RSV RdRp at 2 d and 4 d after lst-miR-92 inhibitor treatment by 67% and 51%, respectively (S8B Fig). These results suggest that lst-miR-92 promotes RSV replication. We then examined whether lst-miR-92 affected RSV titers by measuring RSV *N* levels at various times after injecting lst-miR-92 mimic (mimic-NC was used as control) or lst-miR-92 inhibitor (inhibitor-NC was used as control). RSV *N* transcript concentration increased at 2 d, 4 d, and 6 d after lst-miR-92 mimic treatment by 207%, 83%, and 59%, respectively (Fig 7A). RSV *N* transcript concentration declined at 2 d, 4 d, and 6 d after lst-miR-92 inhibitor treatment by 76%, 61%, and 42%, respectively (Fig 7B). Immunoblot analysis and immunofluorescence assay confirmed the effect of lst-miR-92 on RSV loads (Figs 7A and 7B; S2G).

Next, we aimed at identification of the target genes of lst-miR-92. A total of 81 SBPH genes were predicted as candidate target genes of lst-miR-92 by miRanda, PITA, and RNAhybrid (S3 Table) [52]. Eight SBPH genes were identified as differentially expressed genes (*p*<0.05; log2 fold change <1 or >-1) in viruliferous SBPHs through transcriptomic analysis and small RNA sequencing. Among these genes, *LsFBN2* (encoding fibrillin 2) showed a statistically significant difference (S4 Table). Consequently, we focused on *LsFBN2* for our subsequent experiments. To evaluate whether *LsFBN2* is a direct target of lst-miR-92, Sf9 cells were transfected with luciferase-encoding pGL-Basic3.1 reporter plasmids containing a wild-type *LsFBN2* coding sequence or a mutant *LsFBN2* (*mLsFBN2*) sequence, and luciferase activity was measured (S8C Fig). Luciferase activity was markedly enhanced (4.88-fold) in the cells co-transfected with *LsFBN2*-containing plasmid and the lst-miR-92 mimic relative to the cells co-transfected with *mLsFBN2* and lst-miR-92 mimic (Fig 7C). Similar results were obtained when lst-miR-92 mimic was injected into the viruliferous SBPHs; *LsFBN2* mRNA levels were increased 4.12-fold compared to negative control SBPHs (Fig 7D). We then investigated the LsFBN2 protein levels in lst-miR-92 mimic and mimic-NC-treated nonviruliferous SBPHs at 2, 4, 6, and 8 d. Fibrillin 2 levels showed no significant difference in lst-miR-92 mimic- and mimic-NC-treated nonviruliferous SBPHs at 2 d after treatment (Figs 7E and S2H; S5 Table). At 6 and 8 d after mimic treatment, fibrillin 2 levels in about 60% nonviruliferous SBPHs were specifically activated by lst-miR-92 mimic (Figs 7E and S2H; S5 Table). Additionally, we investigated whether LsRING knockdown affected *LsFBN2* level. RT-qPCR results showed that *LsFBN2* transcript levels in SBPHs at 2, 4, 6, and 8 d after dsLsRING treatment reduced by 39, 46, 64, and 63%, respectively (Fig 7F). These results indicated that *LsFBN2* is the target gene of lst-miR-92 and that fibrillin 2 expression is upregulated by lst-miR-92.

Since fibrillins, extracellular matrix glycoproteins, may play a role in virus replication [53,54], we evaluated whether fibrillin 2 has an effect on RSV replication by injecting third instar viruliferous SBPH nymphs with dsLsFBN2 or dsGFP. RT-qPCR analysis indicated that the *LsFBN2* transcript level was 61% lower in dsLsFBN2-treated nymphs than in dsGFP controls 2 d after injection (Fig 7G). RNAi-mediated repression of *LsFBN2* reduced the transcript levels of RSV *NS3* by 46% and *RdRp* by 57% in SBPHs but had no effect on *N* (Fig 7G). Since RdRp and NS3 play important roles in RSV replication [20,55], we examined whether *LsFBN2* repression affected RSV *N* transcript levels at various times after dsLsFBN2 or dsGFP treatment. RSV *N* transcript levels began to decline at 4 d after dsLsFBN*2* treatment (Fig 7H), which was confirmed by immunoblot analysis and immunofluorescence assay (Figs 7I and 7J; S2I). These results demonstrate that the positive regulation of *LsFBN2* by lst-miR-92 promotes RSV replication and that RSV NS3 is the key player in this promotion.

## Discussion

Viruses function by targeting, modifying, and hijacking nuclear components of the host to promote virus replication [3]. Several DNA and RNA viruses replicate in the nuclei of host cells and use karyopherins (e.g., importins and exportins) to translocate viral components into the nuclei to promote efficient virus replication [10,56,57]. Karyopherins enhance the replication of RNA viruses that replicate in the cytoplasm, including porcine reproductive and respiratory syndrome virus 2, Zika virus, and RSV [8,24]. In addition to using karyopherins, viruses promote replication with ubiquitin-regulated nuclear-cytoplasmic trafficking [3], but the underlying mechanism for trafficking has not been elucidated.

Ubiquitin ligases (E3s) of several protein families function in virus replication in different ways, depending on the virus. RING E3s promote antiviral immunity in response to infection with vesicular stomatitis Indiana virus or herpes simplex virus type 1 [58]. Mind bomb 1, another RING E3, functions in the uncoating of human adenoviruses 2 and 5 (HAdV-2/5) genomes at the nuclear pore complex [33]. CRL E3s are involved in the degradation of p53, enabling efficient HAdV-5 and vaccinia virus replication [35,59] as well as regulation of uncoating and release of influenza A virus particles [34]. K63-linked ubiquitylation functions in subcellular localization of proteins, transcription, DNA repair, and signal transduction [60,61]. Here, we demonstrated the roles of ubiquitylation in virus infections by presenting evidence that K63-linked polyubiquitylation of cytoplasmic RSV nonstructural protein 3 (NS3) at NS3 residue K127 by LsRING translocates NS3 into cell nuclei. NS3 content continued to increase in the cytoplasm of Sf9 cells 12.5–24 h after RSV exposure, while remaining relatively stable in their nuclei until 22.5–24 h (Figs S9, 5A and 5B). Based on these results, and because NS3 contains a predicted nuclear export signal (S10 Fig), we hypothesize that NS3 translocation may be bidirectional.

**Fig 8:**
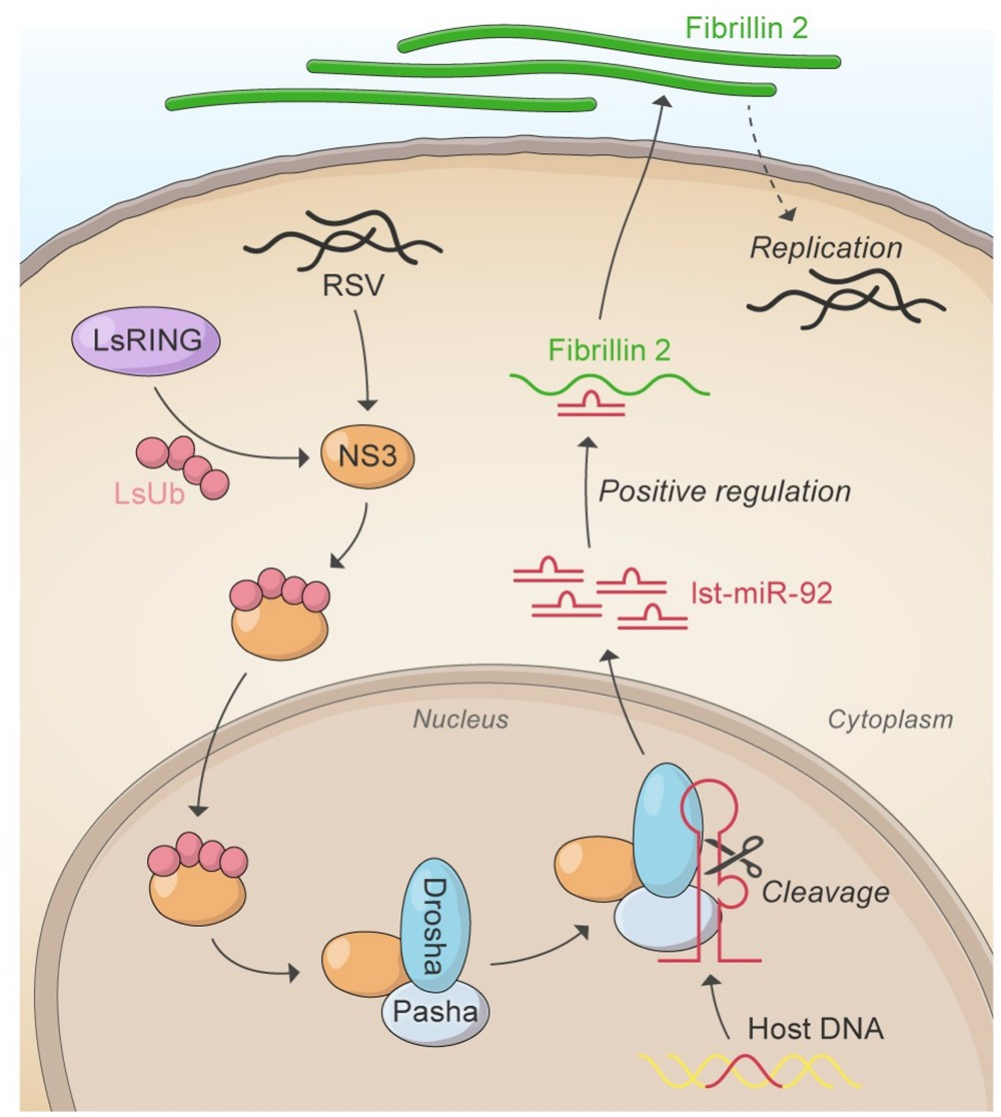
The role of RSV NS3 in RSV replication. Nonstructural protein 3 (NS3) of rice stripe virus (RSV) interacts with small brown planthopper (SBPH) RING E3 (LsRING). LsRING catalyzes the K63-linked ubiquitylation of NS3 (attaining LsUb) and mediates NS3 cytoplasmic-nuclear trafficking. In the SBPH cell nucleus, NS3 regulates lst-pri-miR-92 processing through manipulating the microprocessor complex to induce miR-92 accumulation. Finally, lst-miR-92 positively regulates the gene encoding fibrillin 2, an extracellular matrix protein, thereby indirectly promoting virus replication in the cytoplasm.

The host microRNA (miRNA)-virus interaction plays a role in regulating virus replication [62–69], and the outcome can either be positive (proviral) or negative (antiviral) [51,70]. Host miRNAs bind to virus genomes, preventing virus replication by inhibiting translation [62] or enhancing replication by stabilizing viral RNA [65,71]. Viruses known to engage host miRNAs to manipulate antiviral immune responses and to indirectly regulate virus replication include dengue virus 2 [72], influenza A virus [73], vesicular stomatitis Indiana virus [74], enterovirus A71 [75], and Siniperca chuatsi rhabdovirus [76]. miRNAs are conserved, single-stranded, short (approximately 22 base pairs [bp]-long) non-coding RNAs that regulate gene expression by binding to target messenger RNAs (mRNAs) [77]. Steps in the formation of canonical miRNAs include nuclear processing of a primary miRNA, nuclear export of that precursor miRNA (pre-miRNA), and cytoplasmic processing of pre-miRNA by DICER1, a ribonuclease required for pre-miRNA maturation [49,78]. miRNAs modulate diverse cellular processes including apoptosis, development, differentiation, homeostasis, and immune and stress responses [77,79]. In Asian rice, RSV infection increases the accumulation, for instance lst-miR167, lst-miR171, lst-miR385, lst-miR390, lst-miR398, and lst-miR399 [42,48,80]. Indeed, RSV NS3 recruits pri-miRNAs by interacting with DRB1, a component of the Asian rice miRNA-processing complex [48], and RSV NS3 is already known to hijack the SBPH 26S proteasome by interacting with regulatory particle non-ATPase subunit 3, which attenuates the Asian rice defense response [81]. Our results show that RSV NS3 also manipulates pri-miRNA processing by interacting with the microprocessor complex in the nucleus, thereby upregulating lst-miR-92 in the vector. It has been widely accepted that miRNAs inhibit mRNA transcription via targeting mRNA 3’ untranslated or coding regions. However, activation of gene expression by miRNAs has also been reported. In HEK293 and HeLa cells, Let-7 and the synthetic microRNA miRcxcr4 were associated with AGO2 and FXR1, upregulating translation during cell cycle arrest [82]. In mammals, miRNA-induced upregulated protein expression was also observed in quiescent cells, such as oocytes [83,84]. Competing endogenous RNA (ceRNA) activate PEG10 by acting as a sponge for miR-27-3p and miR-34a-5p [85]. Here, we found that lst-miR-92 targets the coding region of *LsFBN2* and upregulates the expression of fibrillin 2. Furthermore, our results demonstrate that the upregulation of *LsFBN2* by lst-miRn92 promotes RSV replication (see Fig 8 for a graphical summary of all results). We speculate that lst-miR-92 may be associated with translation activators. Alternatively, ceRNA might compete with lst-miR-92 for target binding, thereby upregulating the expression of target genes. Fibrillins are known as large, modular, extracellular matrix glycoproteins that assemble into beaded microfibrils [86]. However, only a few studies have investigated the role of fibrillins in virus replication. For instance, fibrillin 1 supports human immunodeficiency virus 1 replication independent of bone marrow stromal antigen 2 (BST-2/tetherin), an interferon-inducible transmembrane protein [54], and infection by tobacco mosaic virus leads to downregulation of chloroplastic drought-induced stress fibrillin proteins in tobacco plants (*Nicotiana glutinosa* L.) [53]. In addition to their structural role [87], fibrillin microfibrils mediate the signaling pathway of transforming growth factor beta (TGFB) [88,89]. TGFB regulates cell proliferation, apoptosis, matrix homeostasis, and immune responses, which are all closely related to viral infection [90–92]. The balance between cell proliferation and apoptosis is necessary for cellular homeostasis, thereby providing a favorable host cellular environment for virus replication [93,94]. We speculate that fibrillin 2 may indirectly promote RSV replication by regulating cellular homeostasis via the TGFB signaling pathway.

In summary, infection of SBPH with RSV results in K63-linked polyubiquitylation of RSV’s NS3 at residue K127 by LsRING. In turn, ubiquitylation leads to NS3 trafficking from the cytoplasm to the nucleus, where NS3 regulates pri-miR-92 processing through manipulation of the microprocessor complex, resulting in lst-miR-92 accumulation. lst-miR-92 regulates the expression of fibrillin 2, which then promotes RSV replication. Our results highlight the manipulation of intranuclear, cytoplasmic, and extracellular components by a versatile cytoplasmic virus to promote its own replication in an insect vector via the ubiquitin-proteasome system. Our data suggest new avenues for interrupting RSV replication and ultimately reducing dissemination of this important pathogen in one of the world’s most important crops.

## Materials and methods

### Plants, insects, and viruses

Small brown planthoppers (SBPH; Hemiptera: Delphacidae: *Laodelphax striatellus* (Fallén, 1826)), were originally provided by the Jiangsu Academy of Agricultural Sciences, Nánjīng, Jiāngsū Province, China. Viruliferous SBPHs (infected with rice stripe virus [RSV; *Bunyavirales*: *Phenuiviridae*: *Rice stripe tenuivirus*]) and nonviruliferous SBPHs were maintained independently on Asian rice (*Oryza sativa* L.) cultivar 武育粳3 [Wǔyùjīng 3] in glass beakers. Plant materials and insects were cultivated in growth chambers with a 16:8 light/dark photoperiod as described previously [25]. RSV-infected SBPHs were released into glass beakers with Asian rice seedlings (2–3 cm tall), and seedlings were grown until symptoms appeared.

To validate that SBPHs were viruliferous, a single female insect was allowed to feed independently, and parents and offspring were analyzed via dot enzyme-linked immunosorbent assay (dot-ELISA) with virus-specific monoclonal antibodies on a weekly basis [95]. Highly-viruliferous were retained and used in experiments. RSV was isolated from infected Asian rice leaves as described previously [96], and purified virions were stored at -80°C.

### Polymerase chain reaction (PCR), Rapid Amplification of Complementary DNA Ends (RACE), and real-time reverse transcription polymerase chain reaction (RT-qPCR)

Adult SBPHs (n = 5) were collected as a single sample, and total RNA was extracted using TRIzol per the manufacturer’s recommendations (Cat. No. 5346994; Invitrogen, Waltham, MA, USA). PCR primers were designed based on available sequences (S6 Table), and PCR was conducted with LA *Taq* DNA polymerase (Takara, Dàlián, Liáoníng Province, China). 5′ and 3′ Rapid Amplification of Complementary DNA (cDNA) Ends (RACE) was conducted to obtain full-length cDNAs of *E3* and ubiquitin (*Ub*) genes using the SMART RACE cDNA Amplification Kit (Takara Bio USA, Mountain View, CA, USA), as recommended by the manufacturer.

Gene-specific primers (S6 Table) and the SYBR PrimeScript reverse-transcription polymerase chain reaction (RT-PCR) Kit II (Takara) were used to conduct real-time reverse transcription polymerase chain reaction (RT-qPCR) using the Bio-Rad CFX 96 Real-Time PCR system. RT-qPCR conditions were set as follows: 95°C for 30 s followed by 40 cycles at 95°C (10 s) and 60°C (15 s). Transcript abundancy were normalized with SBPH actin (*LsActin*) using the 2^-ΔΔCT^ method [97] and CFX Manager v. 2.1 software (Bio-Rad, Hercules, CA, USA). The mean and standard errors were calculated for at least 3 biologically independent sample sets.

### Antibodies

Polyclonal antisera for recombinant RSV nucleocapsid protein (N) and LsRING (HangZhou HuaAn Biotechnology Co., Ltd., Hángzhōu, Zhèjiāng Province, China) were raised in laboratory mice and European (Chinese white strain) rabbits, respectively. A rabbit polyclonal nonstructural protein anti-RSV NS3 antibody was provided by Dr. Kun Zhang (Yángzhōu University, Jiāngsū Province, China). Commercially available anti-ubiquitin antisera (EPR8830, Cat. No. Ab134953; Abcam, Cambridge, UK) was used to detect SBPH ubiquitin (LsUb) in SBPHs. A rabbit monoclonal anti-Drosha antibody (EPR13046, Cat. No. Ab242147; Abcam) was used to detect SBPH Drosha (LsDrosha). Commercially available K63 linkage-specific anti-ubiquin (Ub) antibody (EPR8590-448, Cat. No. Ab179434; Abcam) was used to detect K63-linked ubiquityl chains in SBPHs. Other antibodies used in this study included: goat anti-mouse immunoglobulin G (IgG) with horseradish peroxidase (HRP) conjugate (Cat. No. CW0102S; CoWin Biosciences [CWBIO], Jiāngsū Province, China), goat anti-rabbit IgG with HRP conjugate (Cat. No. CW0103S; CWBIO), Alexa Fluor 488-labeled goat anti-mouse IgG (Cat. No. 115-545-003; Jackson ImmunoResearch Laboratories, West Grove, PA, USA), Alexa Fluor 555-labeled donkey anti-rabbit IgG (Cat. No. Ab150074; Abcam), rabbit polyclonal anti-glutathione S-transferase (anti-GST; Cat. No. CSB-PA255432; CUSABIO, Wǔhàn, Húběi Province, China), rabbit polyclonal anti-glyceraldehyde-3-phosphate dehydrogenase (anti-GAPDH; Cat. No. Ab157156; Abcam), rabbit polyclonal anti-His tag (Cat. No. 2365; Cell Signaling Technology, Danvers, MA, USA), and normal rabbit anti-IgG (Cat. No. A7016; Beyotime, Shanghai, China).

### DNA constructs and expression vectors

SBPH RING E3 gene (*LsRING*) cDNA fragments were cloned as GST-encoding fusions in plasmid pGEX-4T-1. For yeast two-hybrid (Y2H) assays, the *LsRING* open reading frame (ORF) was cloned in the pGBKT7 vector as bait; RSV N-, SP-, NS2-, NS3-, Nsvc2-, and Nsvc4-encoding ORFs were cloned as prey in pGADT7. To investigate the amino-acid region of NS3 interacting with LsRING, we conducted Y2H analysis with 5 truncated mutants of NS3, including NS3-ΔT1 (amino-acid residues 1–64), NS3-ΔT2 (65–108), NS3-ΔT3 (109–211), NS3-ΔT2N N (65–85), and NS3-ΔT2C (86–108), and seven amino-acid substitutions, including RSV NS3 mut ^GPD^65-67^AAA^, RSV NS3 mut ^DAW^68-70^AAA^, RSV NS3 mut ^TLG^71-73^AAA^, RSV NS3 mut ^SLF^74-76^AAA^, RSV NS3 mut ^KTL^77-79^AAA^, RSV NS3 mut ^IWI^80-82^AAA^, and RSV NS3 mut ^LSH^83-85^AAA^. Sequences encoding NS3 variants were directly inserted into the pGADT7 constructs. For *in vitro* ubiquitylation assays, the ORFs of *LsUb* and seven *LsUb* variants (encoding K6R, K11R, K27R, K29R, K33R, K48R, and K63R LsUb variants) were cloned and constructed into the vector pET28a between *Bam*HI and *Xho*I restriction sites to generate His-tagged recombinant protein expression plasmids. For dual-luciferase reporter assays, *LsFBN2* and *mLsFBN2* were amplified by PCR and then subcloned into the linearized pGL-Basic3.1 reporter vector using *Kpn*I and *Nhe*I restrictions sites. Site-directed mutagenesis of the predicted lst-miR-92 binding site in *LsFBN2* (GGAGTGAGTCGTC to GACCTATCGTTAAT) were performed by the same method as above (for appropriate primers, see S6 Table). For GST pull-down assays, recombinant GST-RING was expressed in *Escherichia coli* M15 and purified as described previously [25]. For overexpression of LsRING in the fall armyworm (*Spodoptera frugiperda* (J. E. Smith, 1797)) Sf9 cells, the *LsRING* ORF was cloned from *Bam*HI/*Eco*RI fragments into a linearized pFastBac HT B vector (Invitrogen) using HT B LsRING primers (S6 Table). All plasmid constructs were generated using the ClonExpress II One-Step Cloning Kit (Vazyme, Nánjīng, Jiāngsū Province, China).

### Immunofluorescence microscopy

To prepare for staining and imaging of tissues, dissected SBPH midguts, ovaries, and salivary glands were fixed for 1 h in 4% paraformaldehyde, rinsed three times in phosphate-buffered saline (PBS; pH 7.4, 10 mM), permeabilized with 1% Triton X-100 in PBS solution (v/v) for 1 h, and blocked as described previously [98]. Samples were then incubated overnight at 4°C with anti-LsUb or anti-RSV N antibodies, washed three times in PBS, and then incubated for 1 h with Alexa Fluor 488 or 555 (Invitrogen) secondary antibodies at a 1:1,000 dilution in PBS. After a brief rinse in PBS, samples were placed onto slides in 4′,6 diamidino-2-phenylindole (DAPI) mounting medium (Solarbio, Beijing, China); images were acquired with a TCS SP8 STED 3X super-resolution microscope (Leica, Wetzlar, Germany) and processed with Leica Application Suite Advanced Fluorescence (LAS AF, Leico) software.

### RNA interference

Double-stranded RNAs (dsRNAs) were synthesized using the TranscriptAid T7 High Yield Transcription Kit (Thermo Fisher Scientific, Waltham, MA, USA) using coding sequences of *LsUb*, *LsRING*, and *LsFBN2*. Approximately 36.8 ng of dsRNAs were injected into second instar nymphs of viruliferous SBPHs with a Nanoliter 2010 injector system (WPI, Sarasota, FL, USA). SBPHs were harvested 3 d after dsRNA injection and analyzed for *LsUb* and *LsRING* transcript abundancy by RT-qPCR; GFP-encoding dsRNA-injected larvae were used as controls.

### Immunoblots and protein detection

Samples collected included the whole bodies, midguts, ovaries, and salivary glands of SBPHs at the indicated stage. Proteins were isolated, separated by 12–20% sodium dodecyl sulfate-polyacrylamide gel electrophoresis (SDS-PAGE), and transferred to polyvinylidene fluoride (PVDF) membranes as described previously [98]. Probes included anti-LsUb (1:1,000 dilution), anti-RSV NS3 (1:500 dilution), anti-RSV N (1:1,000 dilution), anti-GAPDH (1:2,000 dilution), and anti-LsRING (1:100 dilution). Proteins were detected with goat anti-rabbit IgG-conjugated HRP and goat anti-mouse IgG-conjugated HRP antisera at a 1:5,000 dilution. immunoblots were imaged as described previously [98].

Wes (ProteinSimple, San Jose, CA, USA), an alternative to immunoblots, based on principles inherent to capillary electrophoresis and immunoassays, was used as described previously to more precisely measure protein production [99].

### GST pull-down assays

Viruliferous SBPH extracts (1 mg), GST-LsRING (500 μg), and 200 μL of immobilized glutathione-sepharose beads were added to 1,000 μL of pull-down buffer and incubated as described [98]. SBPH extract, incubated with GST, served as a negative control. Beads were washed, and retained proteins were released as described previously [98]. Proteins were separated by SDS—PAGE and detected using anti-GST (MA000031; CUSABIO) and anti-RSV NS3 antibodies.

### Co-immunoprecipitation assays

Total protein from viruliferous SBPHs was collected, lysed, treated with proteinase inhibitor, and centrifuged; protein A/G plus agarose beads were added to the supernatant as described to decrease nonspecific protein binding [100]. Supernatants were then incubated with 2 μg of generic anti-Drosha antibody for 1 h, followed by incubation with protein A/G and agarose beads at 4°C with agitation for 12 h. Protein A/G plus agarose beads were washed, mixed with loading buffer, and denatured by boiling for 5 min; lysates were separated on SDS-PAGE gels and transferred to nitrocellulose membranes as described previously [100]. Membranes were incubated with anti-RSV NS3 or anti-Drosha antibodies for 1.5 h at ambient temperature and then incubated with anti-IgG conjugated with HRP (1:5,000) at 37°C for 1 h. Membranes were imaged as described previously [98], and the relative intensity of protein bands was calculated using Image Lab software v. 5.2.1 (Bio-Rad). Triplicate samples were included in each experiment.

### Yeast 2-hybrid assays

Y2H assays were executed using the Yeastmaker Yeast Transformation System 2 (Takara Bio USA, Mountain View, CA, USA). Briefly, the cDNAs encoding RSV proteins were constructed in pGADT7, and full-length *LsRING* was cloned in the bait plasmid pGBKT7. Positive clones from the library were selected on synthetic defined quadruple-dropout medium and interacting prey constructs were recovered and sequenced [98]. To distinguish positive from false-positive interactions, empty pGADT7 and pGBKT7 were transformed into yeast cells, and ß-galactosidase activity was detected with the HTX Kit (Dualsystems Biotech, Schlieren, Switzerland).

### *In vitro* ubiquitylation of NS3

His-tagged RSV NS3s were provided by Dr. Kun Zhang, whereas LsRING was purified from baculovirus-infected insect cells as described previously [101]. Ubiquitylation reactions were performed at 30°C for 3 h in a volume of 40 μL, containing 40 mM Tris-HCl, pH 7.5, 2 mM dithiothreitol (DTT), 5 mM MgCl_2_, 40 μM ubiquitin (Cat. No. BML-UW9920-0001; Enzo Life Sciences, Farmingdale, NY, USA), 50 nM E1 (Cat. No. BML-UW9410; Enzo), 200–600 nM various E2s (UBCH1, UBCH2, UBCH3, UBCH5a, UBCH5b, UBCH5c, UBCH6, UBCH7, UBCH8, UBCH10, UBCH13/Mms2) (Cat. No. BML-UW9920-0001; Enzo), 5 mM ATP (Cat. No. BML-EW9805; Enzo), 20 μL of RSV NS3, and the purified LsRING as described previously [60]. The reaction was terminated by adding 7 μL of 4× SDS-PAGE sample buffer and boiling for 5 min. Samples were resolved by SDS-PAGE, and the ubiquitylated NS3 was detected by immunoblotting as described [60].

### E3 ubiquitin ligase auto-ubiquitylation

The E3 ligase auto-ubiquitylation assay was performed with an Abcam (ab139469) kit using purified recombinant LsRING protein according to the manufacturer’s instructions. The reaction mixture contained recombinant LsRING, Ubiquitin Ligase Buffer (E3), Ubiquitin, Mg-ATP, Ubiquitin Activating Enzyme Solution (E1), and E2 solution. After incubating at 37°C for 60 min, the mixture was terminated by adding 7 μL of 4× SDS-PAGE sample buffer and boiling for 5 min. Samples were subsequently analyzed by SDS-PAGE and immunoblotting.

### LC-MS/MS analysis and identification of ubiquitylation

Following the *in vitro* ubiquitylation assay, ubiquitylated RSV NS3s were resolved on SDS-PAGE gels and then stained with Coomassie Blue dye. The appropriate NS3-ubiquitin conjugate bands were excised from the gel. Gel samples were destained three times with 125 mM of NH_4_HCO_3_ (Cat. No. A610032-0500; Sangon Biotech, Shanghai, China) in 50% ACN (Cat. No.75-05-8; Biohao, Wǔhàn, Húběi Province, China). Then, proteins were reduced with 10 mM DTT (Cat. No. A300862-0005; Sangon Biotech, Shanghai, China)/125 mM NH_4_HCO_3_ at 56°C for 30 min and alkylated with 55 mM iodoacetamide (Cat. No. 144-48-9; Sangon Biotech, Shanghai, China)/125 mM NH_4_HCO_3_ for 30 min in the dark. After washing twice with 50% ACN, gel pieces were dried in a vacuum centrifuge. Then, 200 ng trypsin (Cat. No. A003702-0001; Sangon Biotech, Shanghai, China) in 25 mM NH_4_HCO_3_ were added, and digestions were maintained at 37°C overnight. After digestion, 0.5% (v/v) formic acid (Cat. No. A503066-0250; Sangon Biotech, Shanghai, China) in 50% ACN was added to extract peptide from the gel pieces. The extraction procedure was repeated, and the three extracts were combined and dried by vacuum centrifugation. The peptide samples were dissolved in 2% ACN/0.1% formic acid and analyzed using a TripleTOF 5600+ mass spectrometer coupled with the SCIEX Eksigent nanoLC System (SCIEX, Framingham, MA, USA). Peptides were loaded onto a C18 trap column (5 μm, 100 μm × 20 mm) and eluted at 300 nL/min onto a C18 analytical column (3 μm, 75 μm × 150 mm) over a 60-min gradient. The two mobile phases were buffer A (2% ACN/0.1% formic acid/98% H_2_O) and buffer B (98% ACN/0.1% formic acid/2% H_2_O). For information dependent acquisition (IDA), survey scans were acquired in 250 ms, and 40 product ion scans were collected at 50 ms per scan. MS1 spectra were collected in the range of 350–1500 m/z, and MS2 spectra were collected in the range of 100–1500 m/z. Precursor ions were excluded from reselection for 15 s.

### Transfection and infection of LsST6-transfected Sf9 cell line

The fall army worm Sf9 insect cell line was provided by Fei Ma (Nanjing Normal University, Nánjīng, Jiāngsū Province, China). Sf9 cells transfected with a LsST6-recombined expression bacmid (GenBank accession: MG589412) were used to study RSV infection and replication based on a previous report [100] as described [98]. LsST6-expressing Sf9 cells were then transfected with bacmids expressing GFP and LsRING using a baculovirus expression system (Bac to Bac, Invitrogen, USA) and established protocols [98,100]. Briefly, 8 x 10^5^ LsST6-expressing Sf9 cells were added to each well of a culture dish. Then, 2 μg of recombinant bacmid-plasmid encoding GFP or LsRING were mixed with 8 μL of Cellfectin II reagent (Invitrogen) and then incubated for 5 h, and the transfection mixture was then removed. At 48 h after transfection, RSV particles (1.5 μg/μL) were added to Sf9 cells using established protocols [58,59]. The RSV-infected Sf9 cells were collected at 20, 20.5, 21, and 24 h after infection, rinsed, fixed, and prepared for immunofluorescence microscopy [98]. Total RNA and protein were extracted from cells for RT-qPCR and immunoblot analyses, respectively [98]. Individual wells within a plate comprised a single replicate, and each treatment contained three replicates.

### Small RNA sequencing

Cloning of small RNAs for Illumina sequencing was performed by Novogene Co., Ltd (Beijing, China). Due to the unavailability of the SBPH genome sequence, the transcriptome of nonviruliferous SBPHs (NCBI Sequence Read Archive Accession no. SRX016334) and 138,858 expressed sequence tags from the planthopper family Delphacidae were used as a genome reference as described [102]. Statistics for the small RNA data sets were obtained using in-house Perl scripts.

### Dual-luciferase reporter assay

Fall armyworm Sf9 cells were seeded at 1.5 × 10^5^ cells per well in 200 μL of Gibco Sf-900 II serum-free media (SFM; Cat. No. 10902088; Fisher Scientific) in 96-well plates 1 d prior to transfection. Transfection of Sf9 cells was performed using FuGENE HD (Promega, Madison, WI, USA). Briefly, 1 μg of pGL-FBN or pGL-mFBN and 20 ng of lst-miR-92 mimic were co-transfected into Sf9 cells; 0.1 μg of pIZT-RLuc vector containing the *Renilla* luciferase gene [103] were used as a reference for insect cells. Co-transfections were repeated three times, and expression concentrations were calculated as mean ± standard error of the mean (SEM). Firefly and *Renilla* luciferases were quantified using the Dual-Glo luciferase assay kit (Promega) as described [104].

### RNA immunoprecipitation assays

RNA immunoprecipitation (RIP) assays were performed to validate the interaction of RSV NS3 and lst-pri-miRNAs using an RNA immunoprecipitation kit (Cat. No. P0101; Geneseed Biotech Co., Ltd., Guǎngzhōu, Guǎngdōng Province, China). SBPH tissues were macerated, lysed, and then incubated with RIP buffer containing magnetic beads conjugated with anti-RSV NS3 antibodies or control IgG (Cat. No. A7016; Beyotime, Shanghai, China). Extracted RNAs were analyzed by RT-qPCR to determine if pri-miRNAs were enriched.

### Proteasome inhibition by MG132

Third instar nymphs of the viruliferous SBPHs were exposed topically to MG132 (50 mM in 0.02% dimethyl sulfoxide [DMSO]) (Cat. No. HY-13259; MedChemExpress, Monmouth Junction, NJ, USA) or 0.5% DMSO and then injected with dsLsRING or dsGFP. Insects were maintained on healthy Asian rice seedlings and then analyzed for transcript or protein concentrations by RT-qPCR or immunoblot analysis, respectively. Each experiment was repeated thrice.

### miRNA mimic and inhibitor injection

A total volume of 36.8 nL of miR-92 mimic (sense 5’-UGACGACUCAUCUUGACUCAAUA-3’, antisense 5’-UUGAGUCAAGAUGAGUCGUCAUU-3’)/ mimic-NC (sense 5’-UUCUCCGAACGUGUCACGUTT-3’, antisense 5’-ACGUGACACGUUCGGAGAATT-3’) or miR-92 inhibitor (5’-UAUUGAGUCAAGAUGAGUCGUCA-3’)/inhibitor-NC (5’-CAGUACUUUUGUGUAGUACAA-3’) (20 μM; Genepharma, Shanghai, China) was delivered into the third-instar viruliferous nymphs by microinjection using a Nanoliter 2010 injector system (WPI, Sarasota, FL, USA).At 2, 4, and 6 d after the injection, SBPHs were collected for RNA or protein isolation. Mimic-NC and inhibitor-NC were designed according to the sequence of *Caenorhabditis elegans* miRNA.

### Statistical analysis

Relative accumulations of mRNAs were compared using the one-way analysis of variance (ANOVA) program in IBM SPSS Statistics software [105]. All data are presented as mean ± SEM, and *p*<0.05 was considered statistically significant.

## Supporting information

S1 Fig Small brown planthopper ubiquitin (LsUb), RING E3 (LsRING), and HECT E3 (LsHECT) functional amino-acid and domain alignments. (A) Schematic representation showing the ubiquitin (Ub) domain of LsUb and deduced amino-acid sequence alignments of Ub from animals of six species (AeUb, DcUb, DmUb, HkUb, HsUb, and LsUb). (B) Schematic representation of LsRING showing the RING domain at residues 1645–1690 and deduced amino-acid sequence alignments of RING of insects from six species (AcRING, ArRING, ClRING, HhRING, LsRING, and NlRING). (C) Schematic representation of LsHECT showing the HECT domain at residues 582–913 and deduced amino-acid sequence alignments of HECT of insects from six species (AfHECT, CsHECT, FoHECT, LsHECT, NlHECT, and ZnHECT). Alignments were constructed using VectorNTI and GeneDoc software. Ae, yellow fever mosquito (*Aedes aegypti* (Linnaeus in Hasselquist, 1762)); Ac, *Atta cephalotes* (Linnaeus, 1758) leafcutter ant; Af, dwarf honey bee (*Apis florea* Fabricius, 1787); Ar, turnip sawfly (*Athalia rosae* (Linnaeus, 1758)); Cl, *Cimex lectularius* Linnaeus, 1758 bedbug; Cs, *Cryptotermes secundus* (Hill 1925) termite; Dc, Asian citrus psyllid (*Diaphorina citri* Kuwayama, 1908); Dm, fruit fly (*Drosophila melanogaster* Meigen, 1830); Fo, western flower thrip (*Frankliniella occidentalis* Pergande, 1895); Hh, brown marmorated stink bug (*Halyomorpha halys* Stål, 1855); Hk, *Hyposmocoma kahamanoa* P. Schmitz & Rubinoff, 2011 moth; Hs, human (*Homo sapiens* Linnaeus, 1758); Ls, small brown planthopper (*Laodelphax striatellus* (Fallén, 1826)); Nl, brown planthopper (*Nilaparvata lugens* (Stål, 1854)); and Zn, Nevada termite (*Zootermopsis nevadensis* Hagen, 1858). Note: The labels for LsUb, LsRING, and LsHECT are shown in bold text.
(TIF)

S2 Fig Protein expression. (A) Relative LsRING, RSV NS3, and ubiquitylated protein expression in viruliferous and nonviruliferous SBPHs. (B) Relative LsRING, RSV N, and ubiquitylated protein expression in viruliferous SBPHs treated with dsLsRING or dsGFP. (C) Relative LsRING and ubiquitylated protein expression in midguts, ovaries, and salivary glands of viruliferous SBPHs and nonviruliferous SBPHs. (D) Relative LsRING, RSV N, and ubiquitylated protein expression in midguts, ovaries, and salivary glands of dsLsRING- or dsGFP-treated viruliferous SBPHs. (E) Relative RSV N expression in viruliferous SBPHs treated with MG132 + dsLsRING, MG132 + dsGFP, and DMSO + dsGFP. (F) Relative of LsRING and RSV NS3 expression in nuclei and cytoplasm of Sf9 cells transfected with LsRING or eGFP expression plasmids 24 h after RSV exposure. (G) Relative RSV N expression in viruliferous SBPHs at 2, 4, and 6 d after miR-92 mimic (mimic-NC) and miR-92 inhibitor (inhibitor NC) treatments. (H) Relative fibrillin 2 expression in nonviruliferous SBPHs at 2, 4, 6, and 8 d after miR-92 mimic or mimic-NC treatment. (I) Relative RSV N expression in viruliferous SBPHs at2, 4, 6, and 8 d after dsGFP or dsLsFBN2 treatment. Protein band intensities were quantified by ImageJ. *p<0.05, **p<0.01 and ***p<0.001; *t*-test analysis, mean ± standard error of the mean (SEM).
(TIF)

S3 Fig Relative intensity of small brown planthopper (SBPH) RING E3 (*LsRING*) messenger RNA (mRNA) levels in midguts, ovaries, and salivary glands of SBPHs treated with double-stranded RNA derived from *LsRING* (dsLsRING). Real-time reverse transcription polymerase chain reaction (RT-qPCR) analysis of *LsRING* in (A) whole bodies (n = 6), (B) midguts (n = 200), (C) ovaries (n = 200), and (D) salivary glands (n = 200) of viruliferous SBPHs treated with double-stranded RNA derived from *GFP* (dsGFP; control) or *LsRING* (dsLsRING). Levels of GAPDH (control), and LsRING were detected by immunoblotting. RT-qPCR data represent mean ± standard error of the mean (SEM) and were analyzed by *t*-test (*p<0.05 and **p<0.01).
(TIF)

S4 Fig Analysis of interactions small brown planthopper ubiquitin (LsUb) and rice stripe virus (RSV) nonstructural (NS) proteins NS3, NS2, Nsvc2, and Nsvc4; disease-specific protein SP; and nucleocapsid protein (N). Evaluation of potential interactions of LsUb and RSV NS3, NS2, Nsvc2, Nsvc4, SP, and N using a yeast two-hybrid assay (Y2H). Yeast cells were co-transformed with LsUb and the viral genes Yeast cells were plated onto quadruple-dropout (QDO) SD-Trp-Leu-His-Ade-20 mM3-AT medium. AD-T + BD-53 (T/53) served as the positive control; AD-T + BD-Lam (T/Lam) served as the negative control.
(TIF)

S5 Fig Auto-ubiquitylation of LsRING. E3 ubiquitin ligase activity of purified recombinant LsRING by auto-ubiquitylation.
(TIF)

S6 Fig Fluorescence-intensity distribution analysis of wild-type NS3 and vNS3^K127^ in Sf9 cells. Analysis of NS3 (green) and DAPI (blue) fluorescence-intensity by LAS X (Leica).
(TIF)

S7 Fig Small brown planthopper Drosha (LsDrosha) amino-acid alignment. Deduced amino-acid sequence alignments of Drosha from animals of four species. Alignments were constructed using VectorNTI and GeneDoc software. Dm, fruit fly (*Drosophila melanogaster* Meigen, 1830); Hs, human (*Homo sapiens* Linnaeus, 1758); Ls, small brown planthopper (*Laodelphax striatellus* (Fallén, 1826)); Nl, brown planthopper (*Nilaparvata lugens* (Stål, 1854)). Note: The labels for LsDrosha are shown in bold text.
(TIF)

S8 Fig Putative folding structure of lst-miR-92 precursors. (A) Real-time reverse transcription polymerase chain reaction (RT-qPCR) analysis of rice stripe virus (RSV) RNA-directed RNA polymerase (*RdRp*) transcript levels in viruliferous small brown planthoppers (SBPHs) at 2 (n = 15), 4 (n = 15), and 6 (n = 15) d after miR-92 mimic or mimic negative control (mimic-NC) treatment. (B) RT-qPCR analysis of RSV *RdRp* transcript levels in viruliferous SBPHs at 2 (n = 15), 4 (n = 15), and 6 (n = 15) d after miR-92 inhibitor or inhibitor-NC treatment. (C) Putative folding structure of lst-miR-92 precursors in SBPHs. Letters within the sequences represent RNA nucleotides, and the lines connecting the letters indicate bonds that form primary and secondary structures. The mature sequence of the wild-type LsFBN2 (LsFBN2) is highlighted in pink, and that of the mutant LsFBN2 (mLsFBN2) is shown in pink.
(TIF)

S9 Fig RSV NS3 protein level in Sf9 cytoplasm. (A) Quantitative analysis of rice stripe virus (RSV) nonstructural protein 3 (NS3) and GAPDH band intensity by ImageJ. (B) Immunoblot analysis of rice stripe virus (RSV) NS3 and GAPDH in cytoplasm of Sf9 cells at 30-min intervals.
(TIF)

S10 Fig Contribution of small brown planthopper E3 (LsRING) and importin (LsImportin) to rice stripe virus (RSV) nonstructural protein 3 (NS3) nuclear translocation, and nuclear export signal predication for NS3. (A) Immunoblot analysis of RSV NS3 and histone H3 in viruliferous small brown plant hoppers treated with double-stranded RNA derived from *GFP* (dsGFP; n = 20), *LsRING* (dsLsRING; n = 20) and *LsImportin5* (dsIPO5; n = 20). (B) A combination of neural networks (NN) and hidden Markov models (HMM) were used to predict the NS3 nuclear export signal (NES), which is an 11-residue sequence, L_18_LENDLTSLSI_28_. NN are computer systems that mimic biologic nervous systems; HMM are formal foundation for making probabilistic models of linear sequence ’labeling’ problems.
(TIF)

S1 Table E3 ubiquitin ligases identified in small brown planthoppers. (DOCX)

S2 Table Distribution of rice stripe virus (RSV) nonstructural viral protein 3 (NS3) in midgut cells of viruliferous small brown planthoppers (SBPHs) after treatment with double-stranded RNAs derived from *GFP* (dsGFP) or *LsRING* (dsLsRING). (DOCX)

S3 Table lst-miR-92 target genes. (DOCX)

S4 Table Differently expressed lst-miR-92 target genes. (DOCX)

S5 Table Change of fibrillin 2 levels in lst-miR-92 mimic- versus mimic-NC-treated nonviruliferous SBPHs at 2, 4, 6, 8 d. (DOCX)

S6 Table Primers used in this study. (DOCX)

S1 File Uncropped blots for Figures. (DOCX)

S2 File Numerical data underlying graphs. (XLSX)
