# Supplementary figures and images for: Polyubiquitylated rice stripe virus NS3 translocates to the nucleus to promote cytosolic virus replication via miRNA-induced fibrillin 2 upregulation
Source: PLoS Pathog. 2024 Mar 20;20(3):e1012112. doi: 10.1371/journal.ppat.1012112 (PMC10984529; doi:10.1371/journal.ppat.1012112)

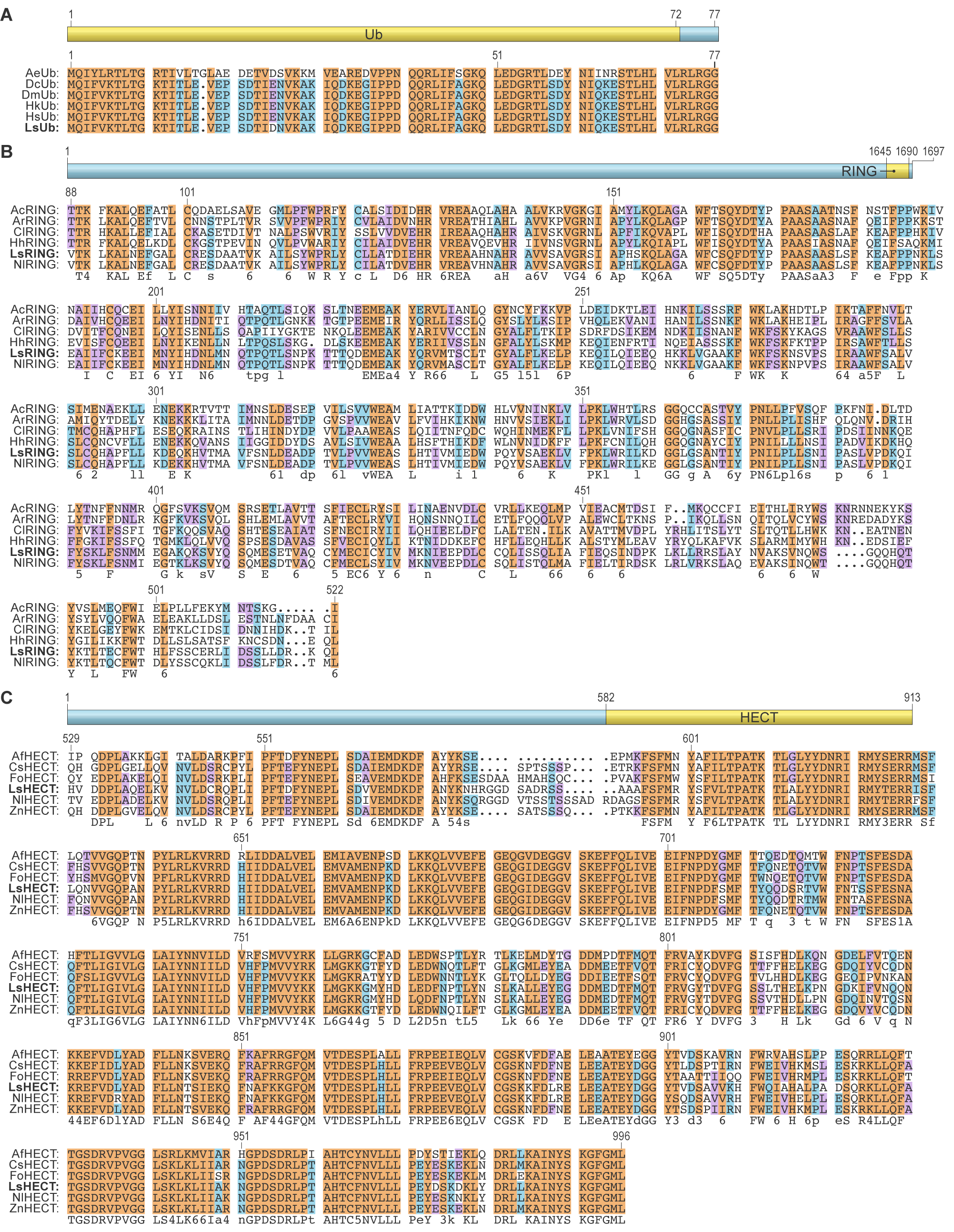

Supplement: S1 Fig — (A) Schematic representation showing the ubiquitin (Ub) domain of LsUb and deduced amino-acid sequence alignments of Ub from animals of six species (AeUb, DcUb, DmUb, HkUb, HsUb, and LsUb). (B) Schematic representation of LsRING showing the RING domain at residues 1645–1690 and deduced amino-acid sequence alignments of RING of insects from six species (AcRING, ArRING, ClRING, HhRING, LsRING, and NlRING). (C) Schematic representation of LsHECT showing the HECT domain at residues 582–913 and deduced amino-acid sequence alignments of HECT of insects from six species (AfHECT, CsHECT, FoHECT, LsHECT, NlHECT, and ZnHECT). Alignments were constructed using VectorNTI and GeneDoc software. Ae, yellow fever mosquito (Aedes aegypti (Linnaeus in Hasselquist, 1762)); Ac, Atta cephalotes (Linnaeus, 1758) leafcutter ant; Af, dwarf honey bee (Apis florea Fabricius, 1787); Ar, turnip sawfly (Athalia rosae (Linnaeus, 1758)); Cl, Cimex lectularius Linnaeus, 1758 bedbug; Cs, Cryptotermes secundus (Hill 1925) termite; Dc, Asian citrus psyllid (Diaphorina citri Kuwayama, 1908); Dm, fruit fly (Drosophila melanogaster Meigen, 1830); Fo, western flower thrip (Frankliniella occidentalis Pergande, 1895); Hh, brown marmorated stink bug (Halyomorpha halys Stål, 1855); Hk, Hyposmocoma kahamanoa P. Schmitz & Rubinoff, 2011 moth; Hs, human (Homo sapiens Linnaeus, 1758); Ls, small brown planthopper (Laodelphax striatellus (Fallén, 1826)); Nl, brown planthopper (Nilaparvata lugens (Stål, 1854)); and Zn, Nevada termite (Zootermopsis nevadensis Hagen, 1858). Note: The labels for LsUb, LsRING, and LsHECT are shown in bold text. (TIF) [file ppat.1012112.s001.tif]

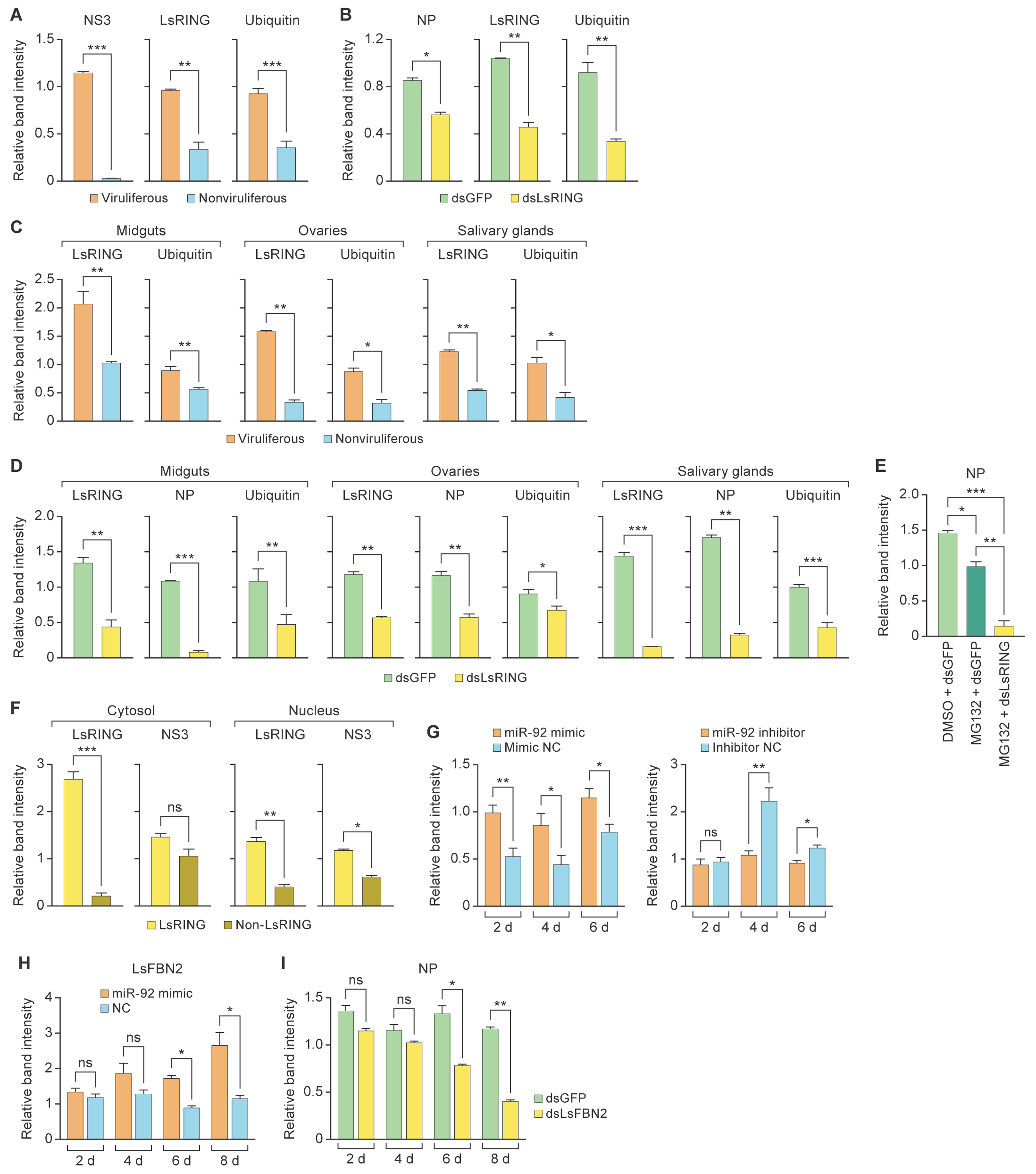

Supplement: S2 Fig — (A) Relative LsRING, RSV NS3, and ubiquitylated protein expression in viruliferous and nonviruliferous SBPHs. (B) Relative LsRING, RSV N, and ubiquitylated protein expression in viruliferous SBPHs treated with dsLsRING or dsGFP. (C) Relative LsRING and ubiquitylated protein expression in midguts, ovaries, and salivary glands of viruliferous SBPHs and nonviruliferous SBPHs. (D) Relative LsRING, RSV N, and ubiquitylated protein expression in midguts, ovaries, and salivary glands of dsLsRING- or dsGFP-treated viruliferous SBPHs. (E) Relative RSV N expression in viruliferous SBPHs treated with MG132 + dsLsRING, MG132 + dsGFP, and DMSO + dsGFP. (F) Relative of LsRING and RSV NS3 expression in nuclei and cytoplasm of Sf9 cells transfected with LsRING or eGFP expression plasmids 24 h after RSV exposure. (G) Relative RSV N expression in viruliferous SBPHs at 2, 4, and 6 d after miR-92 mimic (mimic-NC) and miR-92 inhibitor (inhibitor NC) treatments. (H) Relative fibrillin 2 expression in nonviruliferous SBPHs at 2, 4, 6, and 8 d after miR-92 mimic or mimic-NC treatment. (I) Relative RSV N expression in viruliferous SBPHs at2, 4, 6, and 8 d after dsGFP or dsLsFBN2 treatment. Protein band intensities were quantified by ImageJ. *p<0.05, **p<0.01 and ***p<0.001; t-test analysis, mean ± standard error of the mean (SEM). (TIF) [file ppat.1012112.s002.tif]

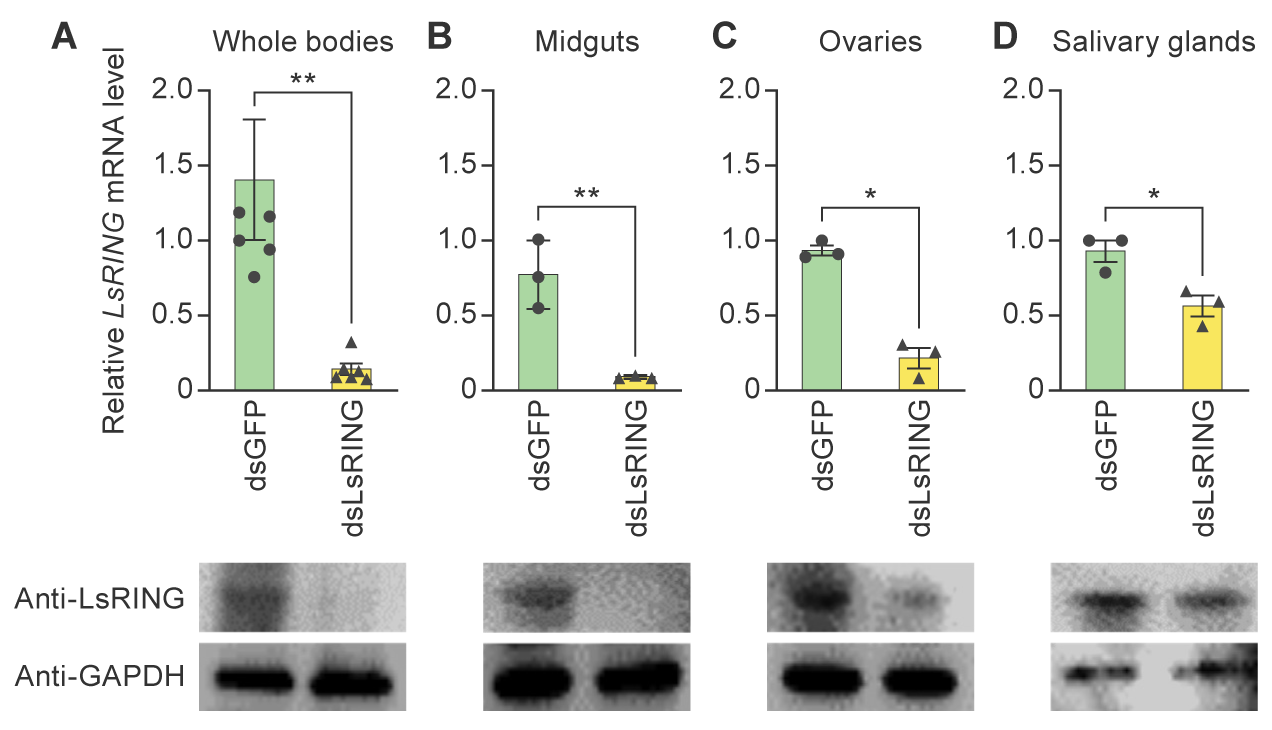

Supplement: S3 Fig — Real-time reverse transcription polymerase chain reaction (RT-qPCR) analysis of LsRING in (A) whole bodies (n = 6), (B) midguts (n = 200), (C) ovaries (n = 200), and (D) salivary glands (n = 200) of viruliferous SBPHs treated with double-stranded RNA derived from GFP (dsGFP; control) or LsRING (dsLsRING). Levels of GAPDH (control), and LsRING were detected by immunoblotting. RT-qPCR data represent mean ± standard error of the mean (SEM) and were analyzed by t-test (*p<0.05 and **p<0.01). (TIF) [file ppat.1012112.s003.tif]

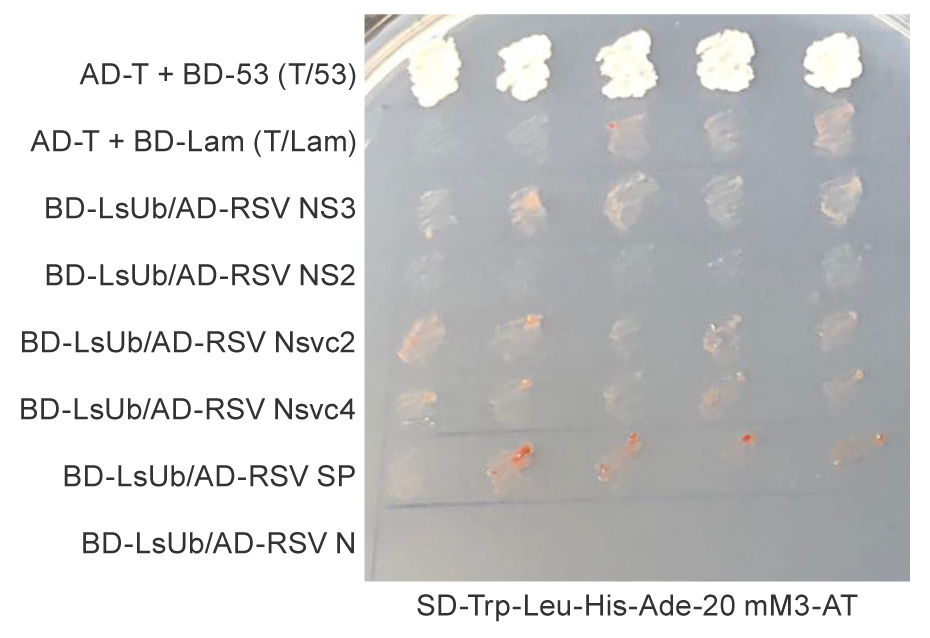

Supplement: S4 Fig — Evaluation of potential interactions of LsUb and RSV NS3, NS2, Nsvc2, Nsvc4, SP, and N using a yeast two-hybrid assay (Y2H). Yeast cells were co-transformed with LsUb and the viral genes Yeast cells were plated onto quadruple-dropout (QDO) SD-Trp-Leu-His-Ade-20 mM3-AT medium. AD-T + BD-53 (T/53) served as the positive control; AD-T + BD-Lam (T/Lam) served as the negative control. (TIF) [file ppat.1012112.s004.tif]

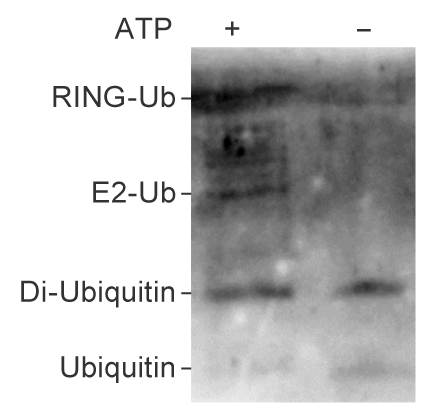

Supplement: S5 Fig — E3 ubiquitin ligase activity of purified recombinant LsRING by auto-ubiquitylation. (TIF) [file ppat.1012112.s005.tif]

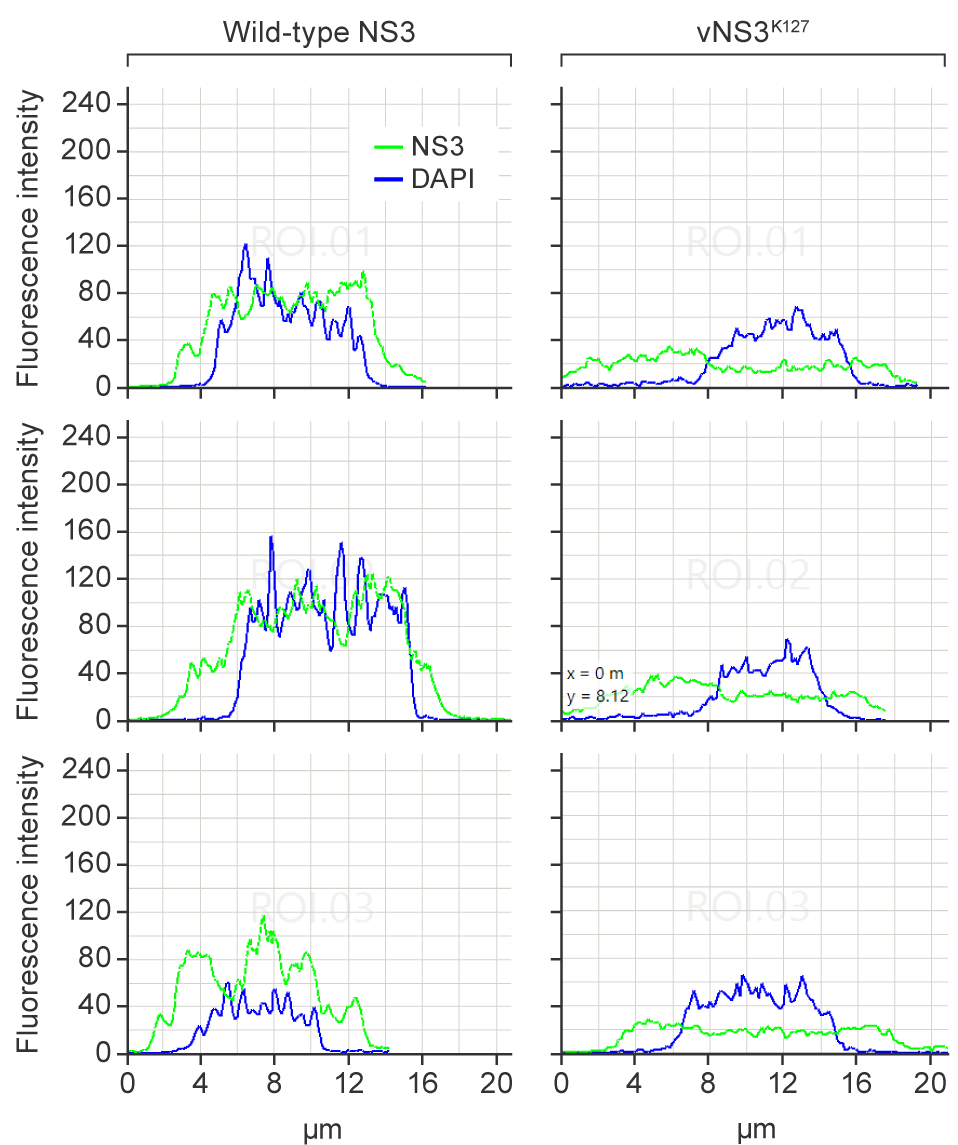

Supplement: S6 Fig — Analysis of NS3 (green) and DAPI (blue) fluorescence-intensity by LAS X (Leica). (TIF) [file ppat.1012112.s006.tif]

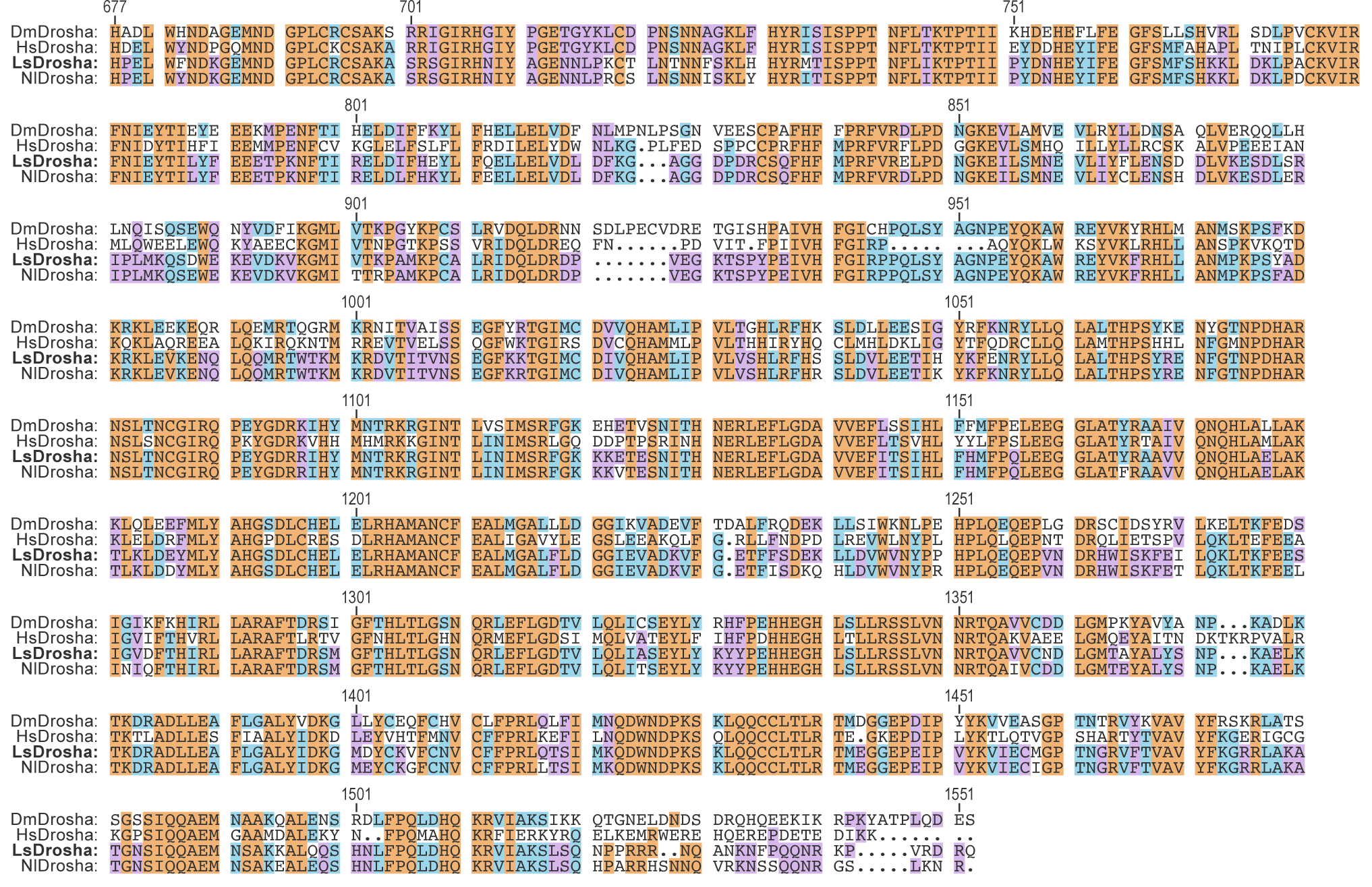

Supplement: S7 Fig — Deduced amino-acid sequence alignments of Drosha from animals of four species. Alignments were constructed using VectorNTI and GeneDoc software. Dm, fruit fly (Drosophila melanogaster Meigen, 1830); Hs, human (Homo sapiens Linnaeus, 1758); Ls, small brown planthopper (Laodelphax striatellus (Fallén, 1826)); Nl, brown planthopper (Nilaparvata lugens (Stål, 1854)). Note: The labels for LsDrosha are shown in bold text. (TIF) [file ppat.1012112.s007.tif]

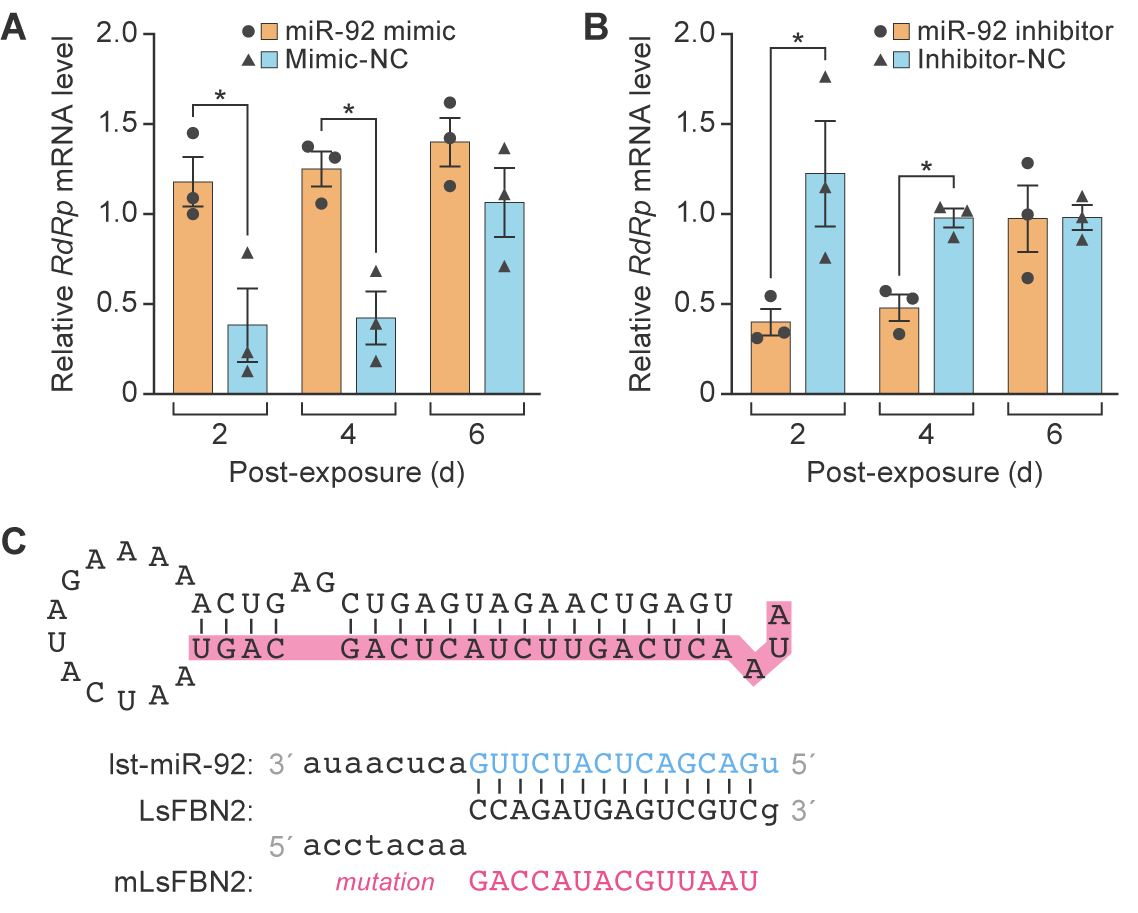

Supplement: S8 Fig — (A) Real-time reverse transcription polymerase chain reaction (RT-qPCR) analysis of rice stripe virus (RSV) RNA-directed RNA polymerase (RdRp) transcript levels in viruliferous small brown planthoppers (SBPHs) at 2 (n = 15), 4 (n = 15), and 6 (n = 15) d after miR-92 mimic or mimic negative control (mimic-NC) treatment. (B) RT-qPCR analysis of RSV RdRp transcript levels in viruliferous SBPHs at 2 (n = 15), 4 (n = 15), and 6 (n = 15) d after miR-92 inhibitor or inhibitor-NC treatment. (C) Putative folding structure of lst-miR-92 precursors in SBPHs. Letters within the sequences represent RNA nucleotides, and the lines connecting the letters indicate bonds that form primary and secondary structures. The mature sequence of the wild-type LsFBN2 (LsFBN2) is highlighted in pink, and that of the mutant LsFBN2 (mLsFBN2) is shown in pink. (TIF) [file ppat.1012112.s008.tif]

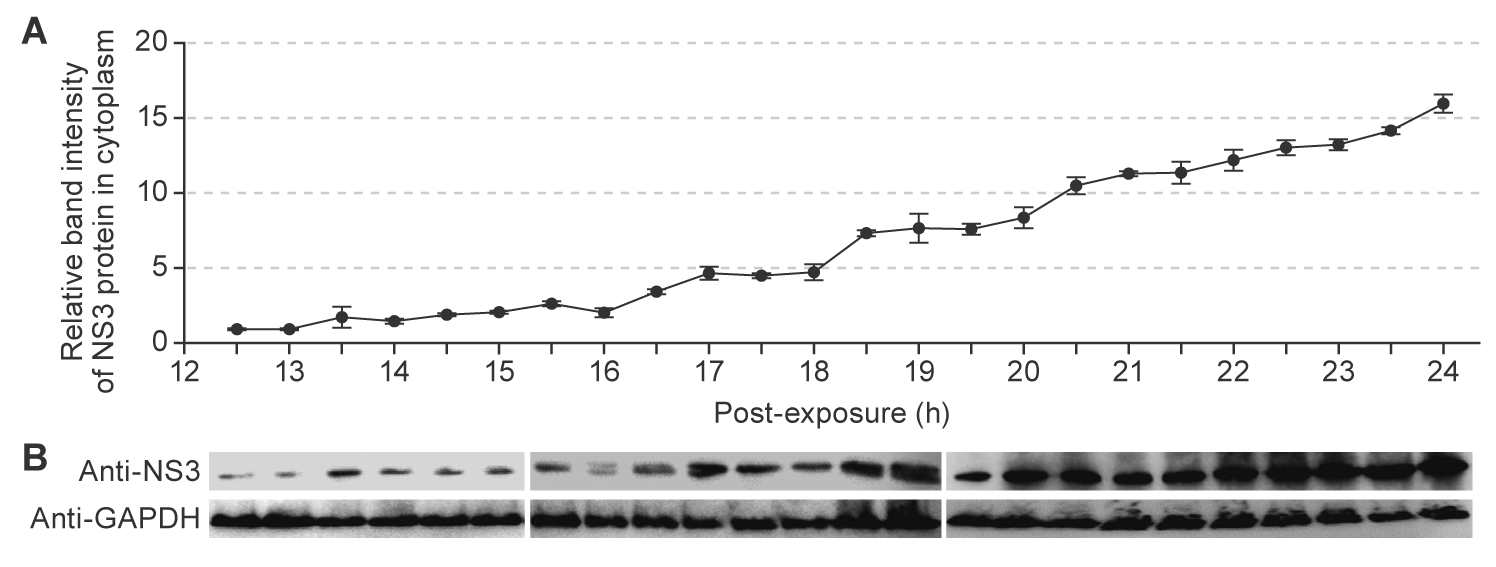

Supplement: S9 Fig — (A) Quantitative analysis of rice stripe virus (RSV) nonstructural protein 3 (NS3) and GAPDH band intensity by ImageJ. (B) Immunoblot analysis of rice stripe virus (RSV) NS3 and GAPDH in cytoplasm of Sf9 cells at 30-min intervals. (TIF) [file ppat.1012112.s009.tif]

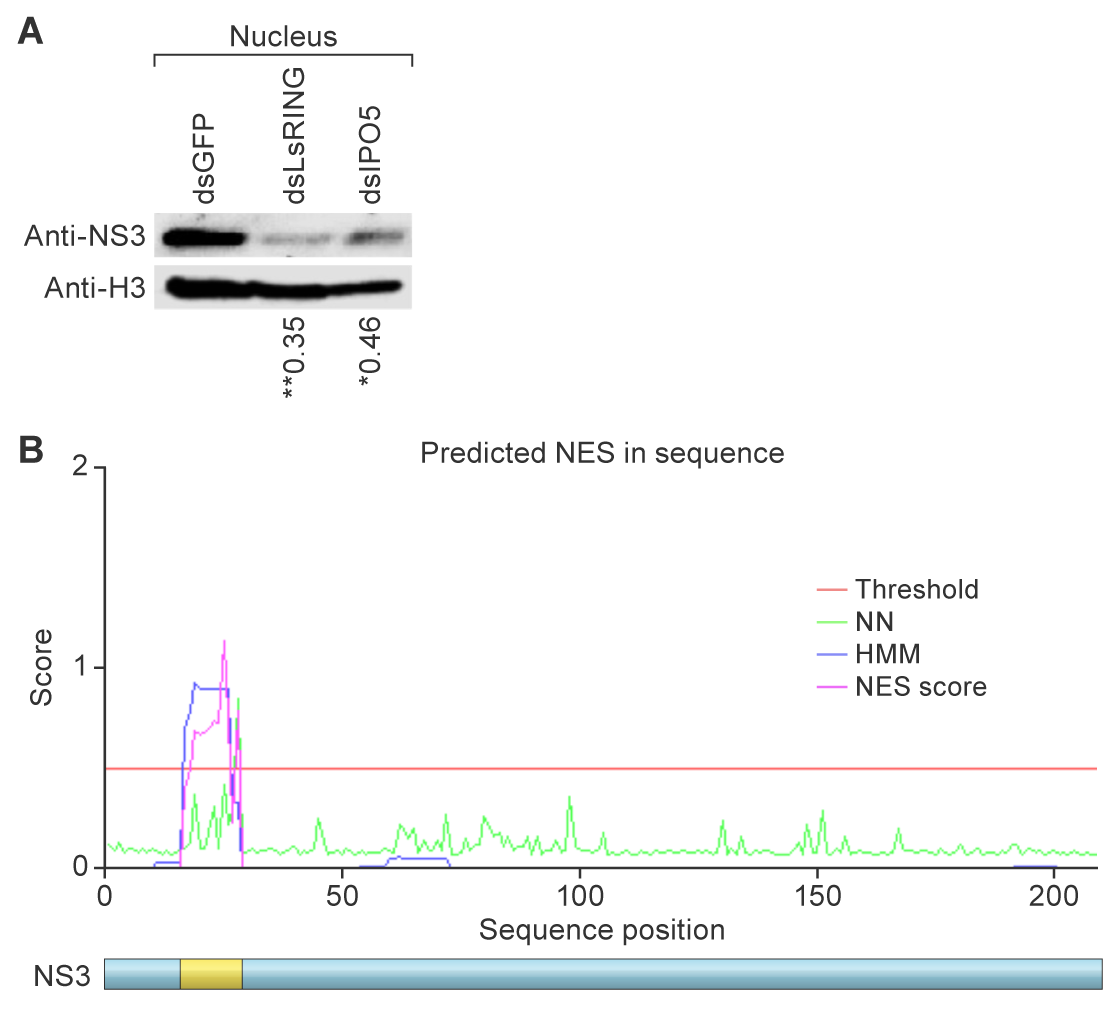

Supplement: S10 Fig — (A) Immunoblot analysis of RSV NS3 and histone H3 in viruliferous small brown plant hoppers treated with double-stranded RNA derived from GFP (dsGFP; n = 20), LsRING (dsLsRING; n = 20) and LsImportin5 (dsIPO5; n = 20). (B) A combination of neural networks (NN) and hidden Markov models (HMM) were used to predict the NS3 nuclear export signal (NES), which is an 11-residue sequence, L18LENDLTSLSI28. NN are computer systems that mimic biologic nervous systems; HMM are formal foundation for making probabilistic models of linear sequence ’labeling’ problems. (TIF) [file ppat.1012112.s010.tif]

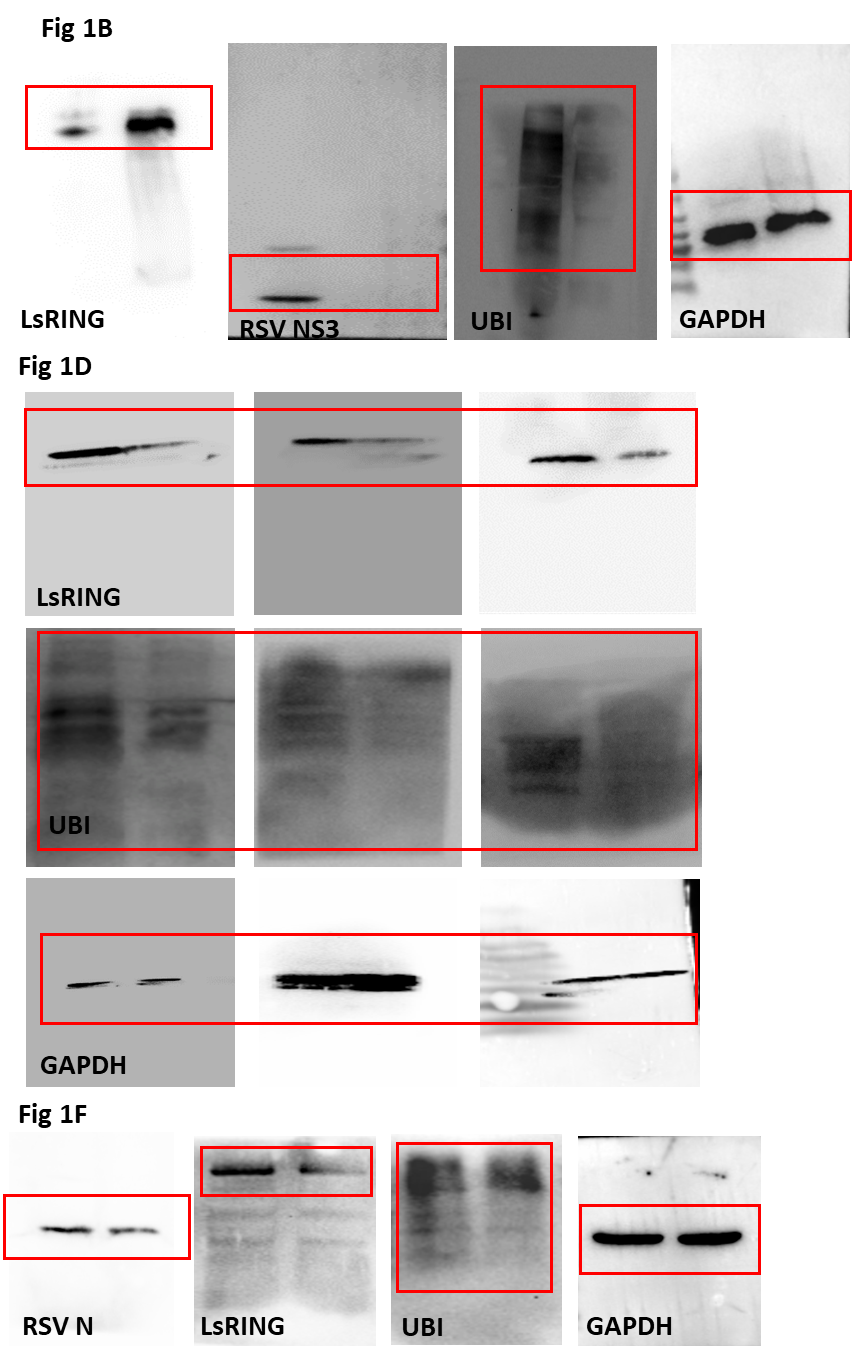


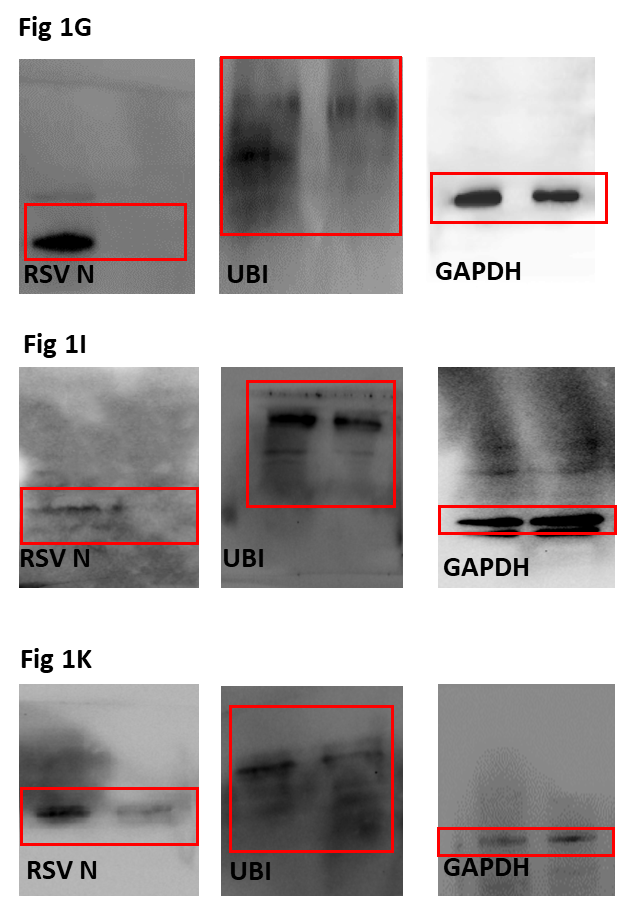


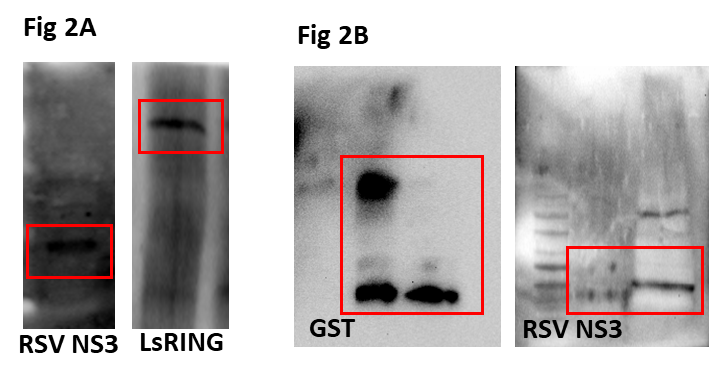


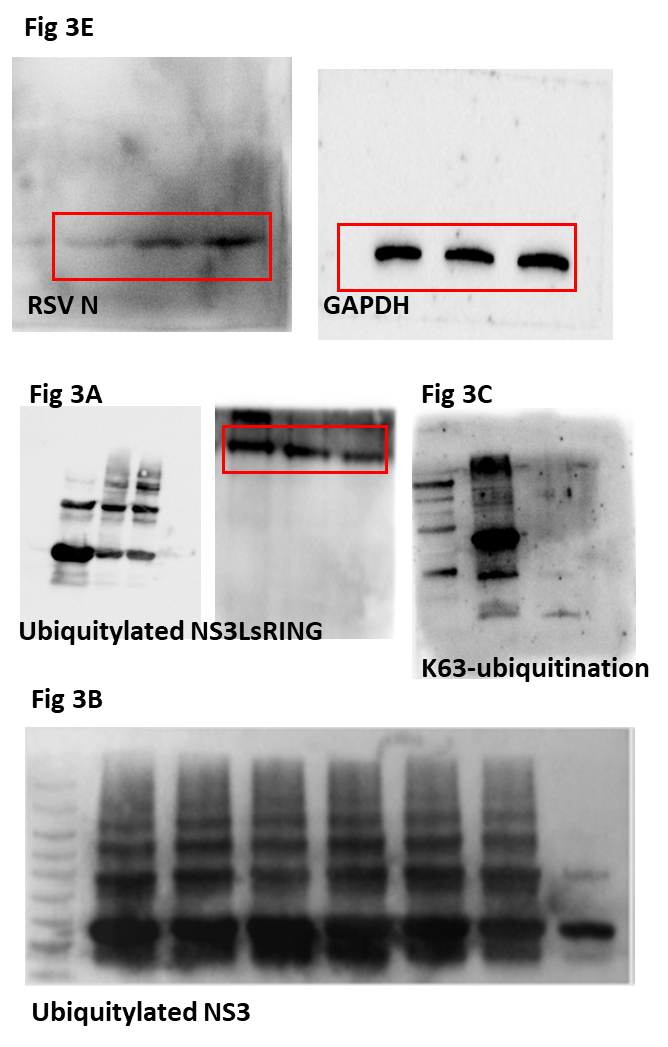


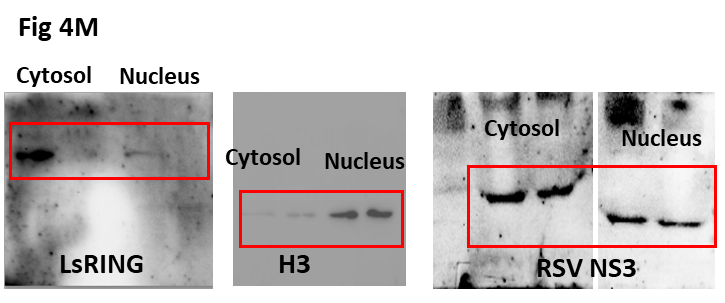


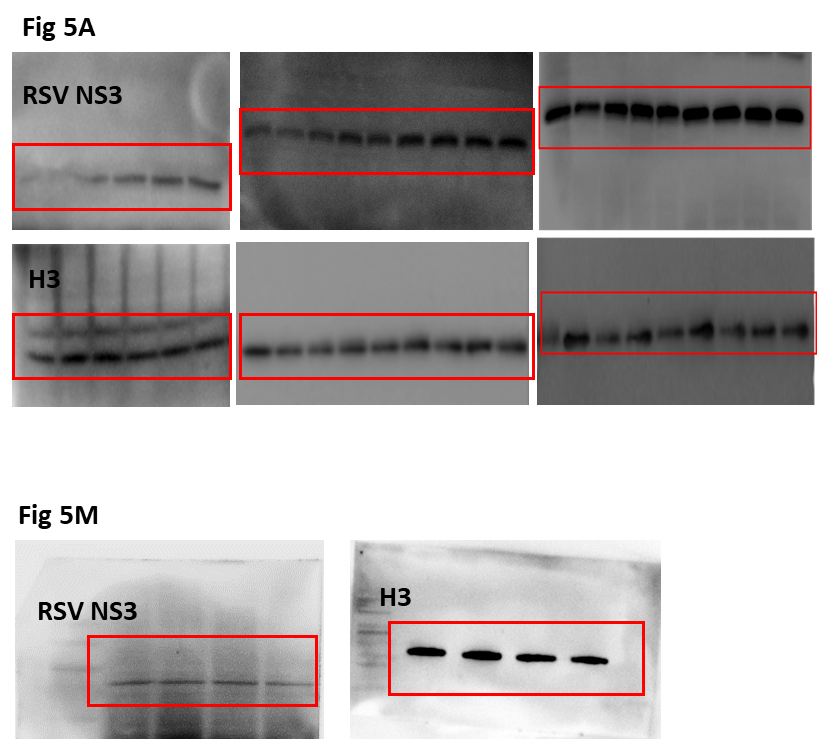


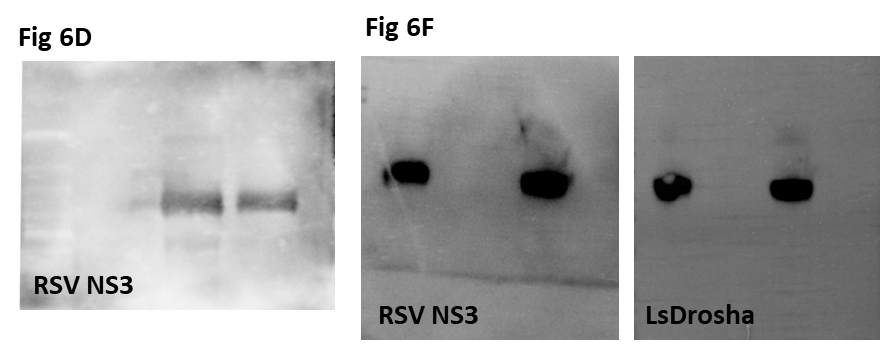


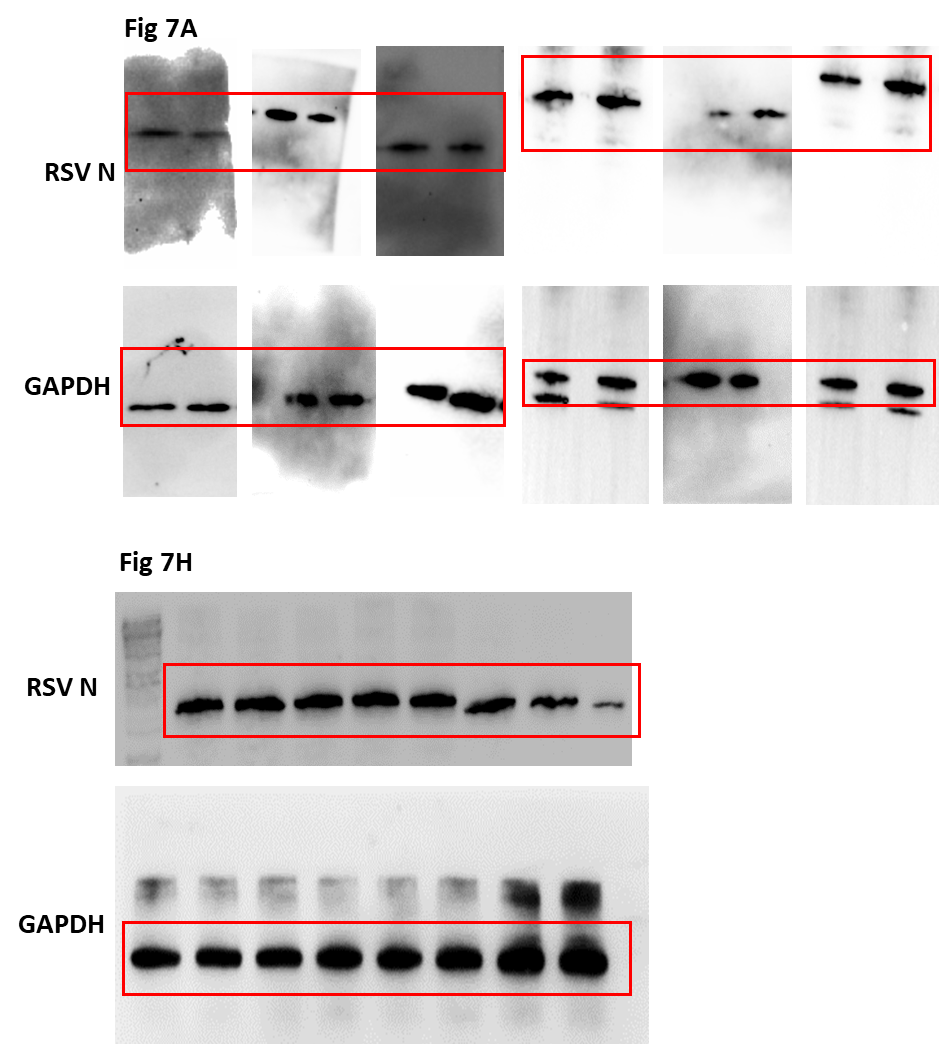


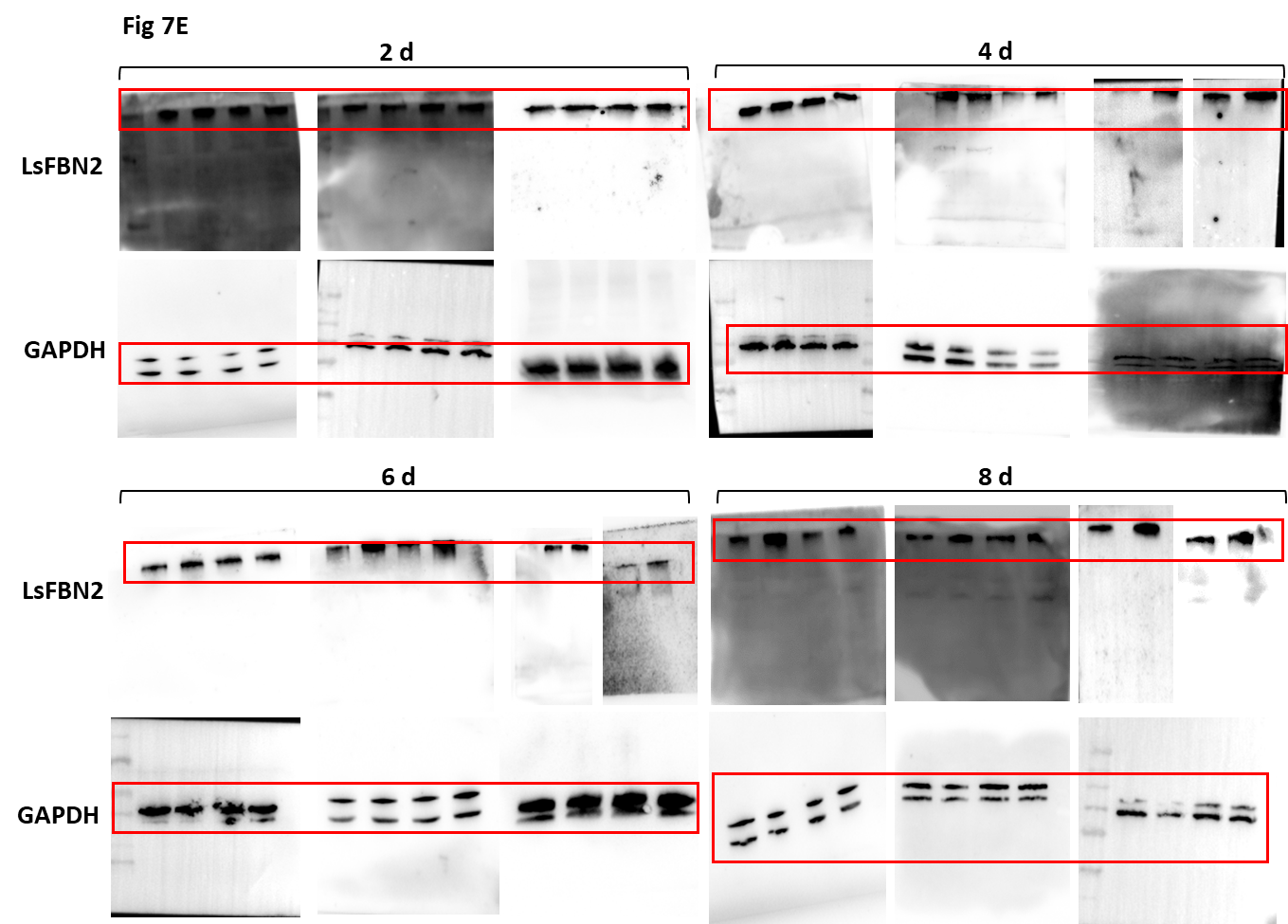


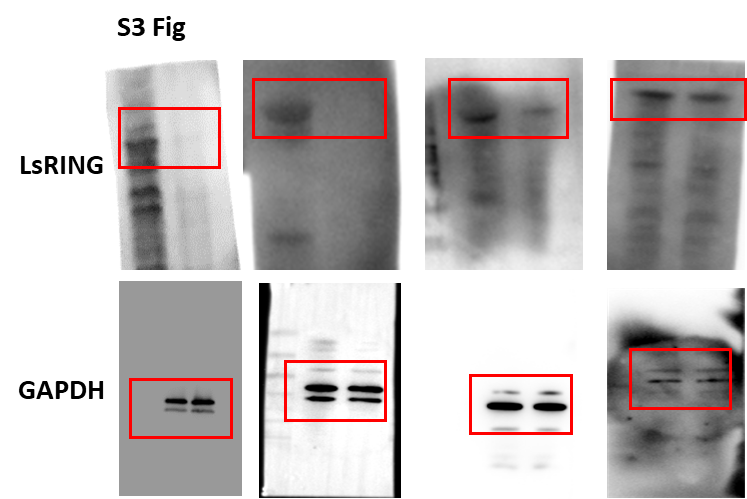

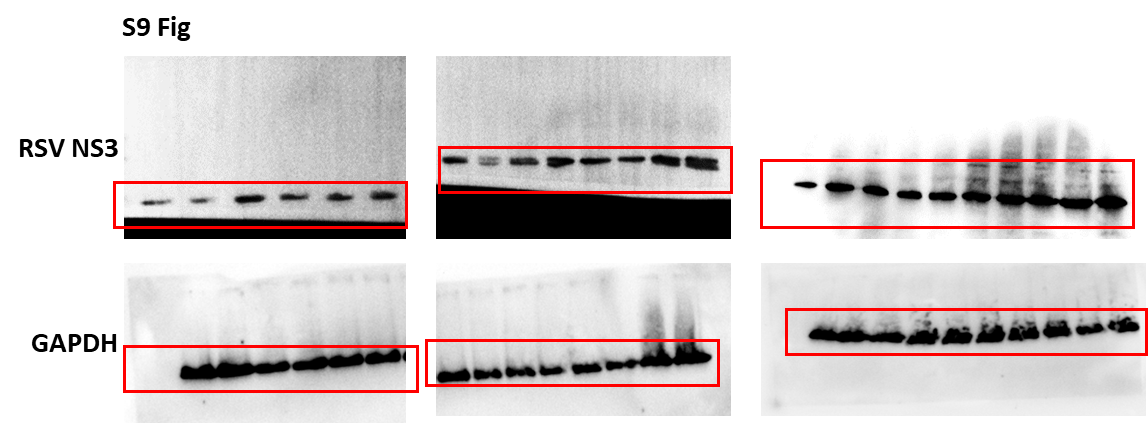

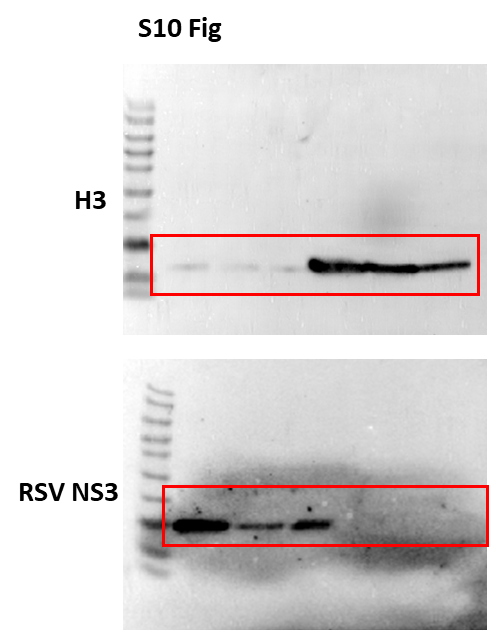

Supplement: S1 File — (DOCX) [file ppat.1012112.s017.docx]
