# Supplementary material for: Polyubiquitylated rice stripe virus NS3 translocates to the nucleus to promote cytosolic virus replication via miRNA-induced fibrillin 2 upregulation
Source: PLoS Pathog. 2024 Mar 20;20(3):e1012112. doi: 10.1371/journal.ppat.1012112 (PMC10984529; doi:10.1371/journal.ppat.1012112)
Supplement: S1 Table — (DOCX) [file ppat.1012112.s011.docx]

## S1 Table. E3 ubiquitin ligases identified in small brown planthoppers.

| Reference Number | Gene Names | Type of Ubiquitin Ligase |
| --- | --- | --- |
| Lstr000506 | E3 ubiquitin-protein ligase *UBR4* | RING |
| Lstr025810 | E3 ubiquitin-protein ligase *HERC1* | HECT |
| Lstr039415 | E3 ubiquitin-protein ligase *PARKIN* | RING-between-RING (RBR) |
| Lstr019578 | Ubiquitin-protein ligase *E3A* | HECT |
| Lstr021251 | E3 ubiquitin-protein ligase *RING* | RING |
| Lstr036369 | E3 ubiquitin-protein ligase *NRDP1* | RING |
| Lstr008291 | E3 ubiquitin-protein ligase *RAD18* | RING |
| Lstr013190 | E3 ubiquitin-protein ligase *RNF220* | RING |
| Lstr039540 | E3 ubiquitin-protein ligase *ZNRF2* | RING |
| Lstr043576 | E3 ubiquitin-protein ligase *MIB1* | RING |
| Lstr004391 | E3 ubiquitin-protein ligase *RNF103* | RING |
| Lstr023832 | E3 ubiquitin-protein ligase *RNF180* | RING |
| Lstr008806 | E3 ubiquitin-protein ligase *RNF19A* | RING-between-RING (RBR) |
| Lstr016860 | E3 ubiquitin-protein ligase *TRIP12* | HECT |
| Lstr024986 | E3 ubiquitin-protein ligase *SHPRH* | RING |
| Lstr039386 | E3 ubiquitin-protein ligase *UFD4* | HECT |
| Lstr018153 | E3 ubiquitin-protein ligase *RNF168* | RING |
| Lstr039365 | E3 ubiquitin-protein ligase *UHRF1* | RING |
| Lstr016572 | E3 ubiquitin-protein ligase *LUBEL* | RING-between-RING (RBR) |
| Lstr036647 | E3 ubiquitin-protein ligase *LRSAM1* | RING |
| Lstr006044 | E3 ubiquitin-protein ligase *UBR3* | RING |
| Lstr013436 | E3 ubiquitin-protein ligase *SIAH1A* | RING |
| Lstr022406 | E3 ubiquitin-protein ligase *DIS1* | RING |
| Lstr038502 | E3 ubiquitin-protein ligase *DIS1* | RING |
| Lstr006503 | E3 ubiquitin-protein ligase *UBR3* | RING |
| Lstr008759 | E3 ubiquitin-protein ligase *RNF181* | RING |
| Lstr013284 | E3 ubiquitin-protein ligase *MYLIP* | RING |
| Lstr030147 | E3 ubiquitin-protein ligase *MDM2* | RING |
| Lstr037747 | E3 ubiquitin-protein ligase *FANCL* | RING |
| Lstr021806 | E3 ubiquitin-protein ligase *HECTD2* | HECT |
| Lstr042622 | E3 ubiquitin-protein ligase *TRIM23* | RING |
| Lstr028021 | E3 ubiquitin-protein ligase *HERC4* | HECT |
| Lstr045908 | E3 ubiquitin-protein ligase *MIB1* | RING |
| Lstr004139 | E3 ubiquitin-protein ligase *AMFR* | RING |
| Lstr025744 | E3 ubiquitin-protein ligase *HERC1* | HECT |
| Lstr039364 | E3 ubiquitin-protein ligase *UBR7* | RING |
| Lstr018574 | E3 ubiquitin-protein ligase *TRIM37* | RING |
| Lstr027704 | E3 ubiquitin-protein ligase *NEDD-4* | HECT |
| Lstr001027 | E3 ubiquitin-protein ligase *HERC2* | HECT |
| Lstr025091 | E3 ubiquitin-protein ligase *HERC1* | HECT |
| Lstr025605 | E3 ubiquitin-protein ligase *HERC1* | HECT |
| Lstr027504 | E3 ubiquitin-protein ligase *HERC4* | HECT |
| Lstr001371 | E3 ubiquitin-protein ligase *HERC2* | HECT |
| Lstr021353 | E3 ubiquitin-protein ligase *HERC1* | HECT |
| Lstr004137 | E3 ubiquitin-protein ligase *AMFR* | RING |
| Lstr041313 | E3 ubiquitin-protein ligase *MARCHF6* | RING |
| Lstr020028 | E3 ubiquitin-protein ligase *TRIM33* | RING |
| Lstr034048 | E3 ubiquitin-protein ligase *RNF185* | RING |
| Lstr027689 | E3 ubiquitin-protein ligase *RNF123* | RING |
| Lstr019368 | E3 ubiquitin-protein ligase *TRIM33* | RING |
| Lstr021631 | E3 ubiquitin-protein ligase *RNF38* | RING |
| Lstr022638 | E3 ubiquitin-protein ligase *RNF165* | RING |
| Lstr017358 | E3 ubiquitin-protein ligase *UBR5* | HECT |
| Lstr020167 | E3 ubiquitin-protein ligase *SH3RF1* | RING |
| Lstr033883 | E3 ubiquitin-protein ligase *RNF157* | RING |
| Lstr017535 | E3 ubiquitin-protein ligase *TRIP12* | HECT |
| Lstr002597 | E3 ubiquitin-protein ligase *MARCHF8* | RING |
| Lstr021607 | E3 ubiquitin-protein ligase *COP1* | RING |
| Lstr031589 | E3 ubiquitin-protein ligase *AMFR* | RING |
| Lstr002956 | E3 ubiquitin-protein ligase *SIAH*-1 | RING |
| Lstr024925 | E3 ubiquitin-protein ligase *HERC1* | HECT |
| Lstr031020 | E3 ubiquitin-protein ligase *TRIM71* | RING |
| Lstr026816 | E3 ubiquitin-protein ligase *TRIM71* | RING |
| Lstr039692 | E3 ubiquitin-protein ligase *TRIM9* | RING |
| Lstr005816 | E3 ubiquitin-protein ligase *UBR3* | RING |
| Lstr032404 | E3 ubiquitin-protein ligase *RING1* | RING |
| Lstr037432 | E3 ubiquitin-protein ligase *CCNB1IP1* | RING |
| Lstr028297 | E3 ubiquitin-protein ligase *TOPORS* | RING |
| Lstr031779 | E3 ubiquitin-protein ligase *RBBP6* | RING |
| Lstr024806 | E3 ubiquitin-protein ligase *ZNF598* | RING |
| Lstr016349 | E3 ubiquitin-protein ligase *SH3RF3* | RING |
| Lstr024685 | E3 ubiquitin-protein ligase SYVN B | RING |
| Lstr021450 | E3 ubiquitin-protein ligase *KCMF1* | RING |
| Lstr024956 | E3 ubiquitin-protein ligase *Iruka* | RING |
| Lstr037492 | E3 ubiquitin-protein ligase *MYCBP2* | RING |
| Lstr042154 | E3 ubiquitin-protein ligase *CBL*-B | RING |
| Lstr004386 | E3 ubiquitin-protein ligase *RNF103* | RING |
| Lstr024318 | E3 ubiquitin-protein ligase *HYD* | HECT |
| Lstr017311 | E3 ubiquitin-protein ligase *HAKAI* | RING |
| Lstr014475 | E3 ubiquitin-protein ligase *UHRF1* | RING |
| Lstr045920 | E3 ubiquitin-protein ligase *MARCHF3* | RING |
| Lstr013069 | Potential E3 ubiquitin-protein ligase *ARIH*-2 | RING-between-RING (RBR) |
| Lstr015175 | E3 ubiquitin-protein ligase *ARIH*-1 | RING-between-RING (RBR) |
| Lstr040987 | E3 ubiquitin-protein ligase *RNF14* | RING-between-RING (RBR) |
| Lstr033827 | E3 ubiquitin-protein ligase *MARCHF3* | RING |
| Lstr035505 | E3 ubiquitin-protein ligase *MARCHF2* | RING |
| Lstr037404 | E3 ubiquitin-protein ligase *RFWD3* | RING |
| Lstr040586 | E3 ubiquitin-protein ligase *RNF144A*-A | RING-between-RING (RBR) |
| Lstr004899 | *Apoptosis-resistant E3 ubiquitin protein ligase 1* | HECT |
| Lstr014164 | E3 ubiquitin-protein ligase *HUWE1* | HECT |
| Lstr015167 | E3 ubiquitin-protein ligase *HECW2* | HECT |
| Lstr024308 | E3 ubiquitin-protein ligase *NEDD*-4 | HECT |
| Lstr044528 | E3 ubiquitin-protein ligase *Su(dx)* | HECT |
| Lstr014314 | E3 ubiquitin-protein ligase *SMURF1* | HECT |
| Lstr032494 | E3 ubiquitin-protein ligase *SMURF2* | HECT |
| Lstr022848 | E3 ubiquitin-protein ligase *HECTD2* | HECT |
| Lstr028706 | E3 ubiquitin-protein ligase *HERC4* | HECT |
| Lstr008299 | E3 ubiquitin-protein ligase *MARCHF5* | RING |
| Lstr014700 | E3 ubiquitin-protein ligase *MIB* | RING |
| Lstr045585 | E3 ubiquitin-protein ligase *MIB2* | RING |
| Lstr013239 | E3 ubiquitin-protein ligase *RNF113A* | RING |
| Lstr044955 | E3 ubiquitin-protein ligase *ZSWIM2* | RING |
| Lstr030409 | E3 ubiquitin-protein ligase *LUBEL* | RING-between-RING (RBR) |
| Lstr041694 | E3 ubiquitin-protein ligase *RNF146* | RING |
| Lstr043628 | E3 ubiquitin-protein ligase *MIB2* | RING |
| Lstr045140 | E3 ubiquitin-protein ligase *MIB2* | RING |
| Lstr010778 | E3 ubiquitin-protein ligase *MSL2* | RING |
| Lstr035661 | E3 ubiquitin-protein ligase *PPIL2* | RING |
| Lstr029152 | E3 ubiquitin-protein ligase *LISTERIN* | RING |
| Lstr017596 | E3 ubiquitin-protein ligase *RNF167* | RING |
| Lstr035603 | E3 ubiquitin-protein ligase *RNF25* | RING |
| Lstr034000 | E3 ubiquitin-protein ligase *Bre1* | RING |
| Lstr028431 | E3 ubiquitin-protein ligase *TTC3* | RING |
| Lstr034089 | *Mitochondrial E3 ubiquitin protein ligase 1* | RING |
