# Supplementary material for: Polyubiquitylated rice stripe virus NS3 translocates to the nucleus to promote cytosolic virus replication via miRNA-induced fibrillin 2 upregulation
Source: PLoS Pathog. 2024 Mar 20;20(3):e1012112. doi: 10.1371/journal.ppat.1012112 (PMC10984529; doi:10.1371/journal.ppat.1012112)
Supplement: S2 Table — (DOCX) [file ppat.1012112.s012.docx]

**S2 Table. Distribution of rice stripe virus (RSV) nonstructural viral protein 3 (NS3) in midgut cells of viruliferous small brown planthoppers (SBPHs) after treatment with double-stranded RNAs derived from *GFP* (dsGFP) or *LsRING* (dsLsRING).**

|  | Number of NS3 antigens detected by immunofluorescence microscopy at 2 d after dsRNA treatments (n*=*30) | | |
| --- | --- | --- | --- |
| Treatment | Nucleus | Cytoplasm | Cytoplasm and nucleus |
| dsGFP | 22 | 7 | 29 |
| dsLsRING | 3 | 23 | 26 |
