# Supplementary material for: Polyubiquitylated rice stripe virus NS3 translocates to the nucleus to promote cytosolic virus replication via miRNA-induced fibrillin 2 upregulation
Source: PLoS Pathog. 2024 Mar 20;20(3):e1012112. doi: 10.1371/journal.ppat.1012112 (PMC10984529; doi:10.1371/journal.ppat.1012112)
Supplement: S3 Table — (DOCX) [file ppat.1012112.s013.docx]

**S3 Table. lst-miR-92 target genes.**

| GenBank Accession Number | Gene Name |
| --- | --- |
| LSTR_LSTR000038 | uncharacterized protein |
| LSTR_LSTR000752 | lachesin |
| LSTR_LSTR001108 | noggin |
| LSTR_LSTR001537 | zinc finger BED domain-containing protein-4 |
| LSTR_LSTR001574 | multicopper oxidase |
| LSTR_LSTR001796 | uncharacterized protein |
| LSTR_LSTR001949 | bromodomain adjacent to zinc finger domain protein |
| LSTR_LSTR002265 | out at first protein |
| LSTR_LSTR002586 | lactosylceramide 4-alpha-galactosyltransferase |
| LSTR_LSTR002855 | uncharacterized protein |
| LSTR_LSTR003272 | uncharacterized protein |
| LSTR_LSTR003312 | ankyrin repeat domain-containing protein |
| LSTR_LSTR003889 | protein FAM60A |
| LSTR_LSTR004472 | Down syndrome cell adhesion molecule |
| LSTR_LSTR004817 | replication protein A 32 kDa subunit |
| LSTR_LSTR005028 | uncharacterized protein |
| LSTR_LSTR005322 | uncharacterized protein |
| LSTR_LSTR005784 | hypothetical protein CINCED |
| LSTR_LSTR005905 | uncharacterized protein |
| LSTR_LSTR005946 | serine incorporator |
| LSTR_LSTR006032 | uncharacterized protein |
| LSTR_LSTR006186 | vitamin D3 receptor |
| LSTR_LSTR006463 | basement membrane-specific heparin sulfate proteoglycan core protein |
| LSTR_LSTR006792 | transient receptor potential channel pyrexia |
| LSTR_LSTR007175 | Armadillo repeat-containing protein |
| LSTR_LSTR007302 | transcription factor RFX |
| LSTR_LSTR007469 | mitochondrial uncoupling protein |
| LSTR_LSTR007761 | protein suppressor of white apricot |
| LSTR_LSTR007889 | fibrillin 2 |
| LSTR_LSTR007894 | centrosomal protein 190 |
| LSTR_LSTR008055 | uncharacterized protein |
| LSTR_LSTR008373 | guanine nucleotide exchange factor DBS |
| LSTR_LSTR008707 | alsin |
| LSTR_LSTR008761 | uncharacterized protein |
| LSTR_LSTR008984 | KAT8 regulatory NSL complex subunit 1 |
| LSTR_LSTR009361 | protein spindle-F |
| LSTR_LSTR009596 | uncharacterized protein |
| LSTR_LSTR009688 | uncharacterized protein |
| LSTR_LSTR009926 | trypsin-2 |
| LSTR_LSTR010560 | trafficking kinesin-binding protein milt |
| LSTR_LSTR010677 | zinc finger protein DZIP1L |
| LSTR_LSTR010732 | thioredoxin-related transmembrane protein |
| LSTR_LSTR010989 | ubiquitin-protein ligase E3C |
| LSTR_LSTR011243 | uncharacterized protein |
| LSTR_LSTR011329 | NECAP-like protein CG9132 |
| LSTR_LSTR011445 | biogenesis of lysosome-related organelles complex 1 subunit 3 |
| LSTR_LSTR011451 | *sn*1-specific diacylglycerol lipase alpha |
| LSTR_LSTR011480 | uncharacterized protein |
| LSTR_LSTR012241 | breast carcinoma-amplified sequence 3 homolog |
| LSTR_LSTR012377 | zinc finger FYVE domain-containing protein |
| LSTR_LSTR012380 | rho GTPase-activating protein |
| LSTR_LSTR012466 | uncharacterized protein |
| LSTR_LSTR012508 | nuclear hormone receptor |
| LSTR_LSTR013142 | protein lethal (2) giant larvae |
| LSTR_LSTR013806 | uncharacterized protein |
| LSTR_LSTR013930 | histone-lysine *N*-methyltransferase |
| LSTR_LSTR014055 | uncharacterized protein |
| LSTR_LSTR014289 | uncharacterized protein |
| LSTR_LSTR014314 | ubinuclein-1 |
| LSTR_LSTR015121 | uncharacterized protein |
| LSTR_LSTR015173 | partitioning defective 3 |
| LSTR_LSTR015216 | DnaJ domain-containing protein [*Wolbachia* endosymbiont of small brown planthoppers] |
| LSTR_LSTR015433 | dentin sialophosphoprotein |
| LSTR_LSTR015629 | acetylcholinesterase |
| LSTR_LSTR015798 | ribosome biogenesis protein |
| LSTR_LSTR016209 | uncharacterized protein |
| LSTR_LSTR016292 | uncharacterized protein |
| LSTR_LSTR016309 | uncharacterized protein |
| LSTR_LSTR016515 | uncharacterized protein |
| LSTR_LSTR016517 | VP1 |
| LSTR_LSTR016602 | neurogenic locus Notch protein |
| LSTR_LSTR016826 | xylosyltransferase oxt-like |
| LSTR_LSTR017102 | asparagine synthetase |
| LSTR_LSTR017275 | transmembrane protein 147 |
| LSTR_LSTR017287 | coiled-coil domain-containing protein 39 |
| LSTR_LSTR017379 | UDP-glucuronosyltransferase |
| LSTR_LSTR017409 | uncharacterized protein |
| LSTR_LSTR017423 | polyribonucleotide nucleotidyltransferase 1 |
| LSTR_LSTR017457 | uncharacterized protein |
| LSTR_LSTR017464 | eIF-2-alpha kinase GCN2 |
| LSTR_LSTR017710 | uncharacterized protein |
