# Supplementary material for: Polyubiquitylated rice stripe virus NS3 translocates to the nucleus to promote cytosolic virus replication via miRNA-induced fibrillin 2 upregulation
Source: PLoS Pathog. 2024 Mar 20;20(3):e1012112. doi: 10.1371/journal.ppat.1012112 (PMC10984529; doi:10.1371/journal.ppat.1012112)
Supplement: S4 Table — (DOCX) [file ppat.1012112.s014.docx]

**S4 Table. Differently expressed lst-miR-92 target genes.**

| GenBank Accession Number | Gene Name | *p*-value |
| --- | --- | --- |
| LSTR_LSTR007889 | fibrillin 2 | 0.001739111 |
| LSTR_LSTR010560 | trafficking kinesin-binding protein milt | 0.003436426 |
| LSTR_LSTR010677 | zinc finger protein DZIP1L | 0.003730383 |
| LSTR_LSTR014055 | uncharacterized protein | 0.002513489 |
