# Supplementary material for: Polyubiquitylated rice stripe virus NS3 translocates to the nucleus to promote cytosolic virus replication via miRNA-induced fibrillin 2 upregulation
Source: PLoS Pathog. 2024 Mar 20;20(3):e1012112. doi: 10.1371/journal.ppat.1012112 (PMC10984529; doi:10.1371/journal.ppat.1012112)
Supplement: S5 Table — (DOCX) [file ppat.1012112.s015.docx]

## S5 Table. Change of fibrillarin 2 levels in lst-miR-92 mimic- versus mimic-NC-treated nonviruliferous SBPHs at 2, 4, 6, 8 d.

| Treatment (mimics) | Time  (d) | Number of Activated Samples | Number of Inhibited Samples | Number of Samples Without Significant Differences |
| --- | --- | --- | --- | --- |
| miR-92/NC | 2 | 0 | 0 | 10 |
| miR-92/NC | 4 | 3 | 1 | 6 |
| miR-92/NC | 6 | 5 | 1 | 4 |
| miR-92/NC | 8 | 6 | 1 | 3 |
