# Supplementary material for: Polyubiquitylated rice stripe virus NS3 translocates to the nucleus to promote cytosolic virus replication via miRNA-induced fibrillin 2 upregulation
Source: PLoS Pathog. 2024 Mar 20;20(3):e1012112. doi: 10.1371/journal.ppat.1012112 (PMC10984529; doi:10.1371/journal.ppat.1012112)
Supplement: S6 Table — (DOCX) [file ppat.1012112.s016.docx]

## S6 Table. Primers used in this study.

| Gene/miRNA | Primer Designation | Sequence |
| --- | --- | --- |
| RSV *NP* | q-RSV-F | TGAAAGTGGCGGCTGGAA |
|  | q-RSV-R | CCACCGAGGACACTATCCCATA |
| RSV *RdRp* | q-RdRp-F | ATAGGCAGTCCAGAATCAGGGTAT |
|  | q-RdRp-R | CGTTCAGTAGTATGAGGAGTGTCCAA |
| RSV *NS3* | q-NS3-F | CATCGTCTGTGGGTTCTGTG |
|  | q-NS3-R | GGAAGGGTGCCTAGATGAATG |
| *LsActin* | Actin-F | GTCTCACACACAGTCCCCATCTATG |
|  | Actin-R | TCGGTCAAGTCACGACCAGC |
| *GFP* | GFP-F | AAGGGCGAGGAGCTGTTCACCG |
|  | GFP-R | CAGCAGGACCATGTGATCGCGC |
|  | T7GFP-F | TAATACGACTCACTATAGGGAAGGGCGAGGAGCTGTTCACCG |
|  | T7GFP-R | TAATACGACTCACTATAGGG CAGCAGGACCATGTGATCGCGC |
| *LsRING* | ds-RING-F | GATGCTCTCACCTACACGCA |
|  | ds-RING-R | TGCCAGCAAACGGTAGTAGG |
|  | T7-RING-F | TAATACGACTCACTATAGGGATGCTCTCACCTACACGCA |
|  | T7-RING -R | TAATACGACTCACTATAGGTGCCAGCAAACGGTAGTAGG |
|  | HTB-RING-F | TTTCAGGGCGCCATGGGATCCATGATGAACAAGAAAGACTCAGT |
|  | HTB-RING-R | TCGACGTAGGCCTTTGAATTCTCAATTAGCCCAGTAGTCGT |
|  | q-RING-F | TCCACCAATCATCCACTG |
|  | q-RING-R | AAGAGGACAACTTCATAACC |
| *LsFBN2* | FBN2-F | CACCAGTTCTCCGGTCTGTC |
|  | FBN2-R | CACACCTGCTAGCTTTCCCA |
|  | PGL-FBN2-F | CACCAGTTCTCCGGTCTGTC |
|  | PGL-FBN2-R | CACACCTGCTAGCTTTCCCA |
|  | PGL-mFBN2-F | GACCATACGTTAATGATACGAGAACGAGTGTGCCACC |
|  | PGL-mFBN2-R | ATCATTAACGTATGGTCTTGTAGTAGATGTGCTGCAGAAATCC |
| miRNA | q-18s-F | GTAACCCGCTGAACCTCC |
|  | q-18s-F | GTCCGAAGACCTCACTAAATCA |
|  | q-miR-79 | TTGGTCGCAGTGGAGCTTTCAA |
|  | q-miR-80 | GGCCATTGTCTGATAGGTACCATT |
|  | q-miR-75 | GGCCTTATTTGATCTCTTGTTAATACA |
|  | q-miR-186 | GGCTGGTATTTTTCACATCAAACTCA |
|  | q-miR-147 | TGGTAACTACCAGCACAACCTCA |
|  | q-miR-177 | GGCGGATCTTCCTGTAGAATTGTT |
|  | q-miR-36 | TAATAACGGTCTACTGTAGCCCAGA |
|  | q-miR-110 | GGCTCAAGTCTTACCAATCAATATGT |
|  | q-miR-122 | GGCCTGTAGAATAATCAAAATCTGTG |
|  | q-miR-315-5p | GGTTTTGATTGTTGCTCAGAAAGC |
|  | q-miR-168 | GAAAGTCCCCACTTAGACGTAGA |
|  | q-miR-117 | GGGAAGATGAGTACGATGACC |
|  | q-miR-92 | GTGACGACTCATCTTGACTCAATA |
|  | q-miR-99 | GCTTATCTTTATGCAGTTGGCAATGA |
|  | q-miR-8-5p | CATCTTACCGGGCAGCATTAGA |
|  | q-miR-276a-5p | CAGCGAGGTATAGAGTTCCTACG |

*GFP*, green fluorescent protein gene; *LsActin*, small brown planthopper (SBPH) actin gene; *LsFBN2*, SBPH fibrillin 2 gene; *LsRING*, SBPH RING E3; *N*, nucleocapsid protein gene; *NS3*, nonstructural protein 3 gene; *RdRp*, RNA-directed RNA polymerase gene; RSV, rice stripe virus.
